# Droplet-Microfluidic-Based Promoter Engineering and Expression Fine-Tuning for Improved Erythromycin Production in *Saccharopolyspora erythraea* NRRL 23338

**DOI:** 10.3389/fbioe.2022.864977

**Published:** 2022-04-04

**Authors:** Kaiyue Yun, Yue Zhang, Shixin Li, Yan Wang, Ran Tu, Hao Liu, Meng Wang

**Affiliations:** ^1^ College of Biotechnology, Tianjin University of Science and Technology, Tianjin, China; ^2^ Key Laboratory of Systems Microbial Biotechnology, Chinese Academy of Sciences, Tianjin, China; ^3^ Tianjin Institute of Industrial Biotechnology, Chinese Academy of Sciences, Tianjin, China

**Keywords:** promoter engineering, Saccharopolyspora erythraea, droplet-based microfluidics, high-throughput screening, erythromycin, gene overexpression, production improvement

## Abstract

Erythromycin is a clinically important drug produced by the rare actinomycete *Saccharopolyspora erythraea*. In the wide-type erythromycin producer *S. erythraea* NRRL 23338, there is a lack of systematical method for promoter engineering as well as a well-characterized promoter panel for comprehensive metabolic engineering. Here we demonstrated a systematical promoter acquiring process including promoter characterization, engineering and high-throughput screening by the droplet-microfluidic based platform in *S. erythraea* NRRL 23338, and rapidly obtained a panel of promoters with 21.5-fold strength variation for expression fine-tuning in the native host. By comparative qRT-PCR of *S. erythraea* NRRL 23338 and a high-producing strain S0, potential limiting enzymes were identified and overexpressed individually using two screened synthetic promoters. As a result, erythromycin production in the native host was improved by as high as 137.24 folds by combinational gene overexpression. This work enriches the accessible regulatory elements in the important erythromycin-producing strain *S. erythraea* NRRL 23338, and also provides a rapid and systematic research paradigm of promoter engineering and expression fine-tuning in the similar filamentous actinomycete hosts.

## Introduction

Actinomycetes produce plenty of bioactive natural products from their secondary metabolism with various applications, including antibacterials, antifungals, anthelmintics, and anticancer agents (Pham et al., 2019). These secondary metabolites are usually biosynthesized by a series of enzymes or enzyme complexes encoded by biosynthetic gene clusters (BGCs). For example, erythromycin is an clinically important macrolide produced as a secondary metabolite by the filamentous actinomycete *Saccharopolyspora erythraea*, and its biosynthesis is controlled by a type I polyketide synthases (PKSs) system encoded by the *ery* BGC (Park et al., 2010). Genome sequencing and meta-genomic mining have revealed that actinomycete genomes usually contain dozens of BGCs which are far from identification and characterization, indicating their tremendous biosynthetic potential to produce new natural products (Rutledge and Challis, 2015). However, native actinomycete hosts usually show low productivities of target products that are far from ideal for large-scale production (Li et al., 2019). The fast-developing synthetic biology provides many solutions to address this problem, such as optimizing the precursor availability, balancing intracellular cofactors, manipulating pathway-related genes, and enhancing metabolite efflux (Bu et al., 2021). To achieve these purposes, promoter engineering and gene overexpression are the most direct strategies to coordinate target gene transcription, and strong promoters are often used to increase expression of rate-limiting enzymes (Zhu et al., 2020; Bu et al., 2021). Generally speaking, high promoter activities can contribute to improved expression level of target genes, but it will also induce accompanying metabolic burdens or even genetic instability in host cells (Sleight et al., 2010; Pasini et al., 2016). Thus, in practical application, appropriate promoters are preferred to balance biosynthetic efficiency and stability and vitality of the producer.

Promoters of different strengths can be used to precisely tune the expression levels of different genes in a biosynthetic pathway so that the metabolic flux in the cell factory can be optimally coordinated and contribute to a high production (Zhu et al., 2020). Hence, well-characterized promoters of different strengths are essential tools for metabolic engineering and synthetic biology in actinomycetes. For many model species of *Streptomyces* genus, such as *S. coelicolor*, *S. lividans* and *S. albus*, a set of constitutive promoters with varied strengths have been identified to facilitate expression fine-tuning and heterologous biosynthesis in these hosts (Shao et al., 2013; Li et al., 2015; Luo et al., 2015). However, for the non-*Streptomyces* actinomycetes, which are also known as rare actinomycetes (Lü et al., 2020), such as *S. erythraea*, well-characterized promoters are still insufficient. The genome sequenced wild-type strain *S. erythraea* NRRL 23338 with low erythromycin production is extensively used as the starting strain and expression host to obtain higher erythromycin yield through random and rational engineering (Oliynyk et al., 2007). Till now, although some advances about BGC manipulation and yield promotion in *S. erythraea* NRRL 23338 have been reported, there are only a few promoters, including *p*_ermE*_, *p*_*KasO*_, *p*_*j23119*_, were employed in this strain (Kirm et al., 2013; Liu et al., 2019). No systematical and easy method to mine and engineer promoters has been reported in *S. erythraea* NRRL 23338, and also there is a lack of a well-characterized promoter panel of different strengths for comprehensive metabolic engineering and expression fine-tuning in this strain.

In a previous work, we have developed a droplet-based microfluidic platform for *Streptomyces*, which is applicable to promoter engineering and protein-driven high-throughput screening (Tu et al., 2021). In this study, we systematically performed promoter characterization, engineering and high-throughput screening in *S. erythraea* NRRL 23338 using the droplet-microfluidic based platform. A collection of synthetic promoters with 21.5-fold strength variation were successfully screened out by fluorescence-activated droplet sorting (FADS) from two random mutagenesis promoter libraries. Potential limiting synthases in erythromycin biosynthesis in *ery* cluster were identified by comparative qPCR between the low-producing wild-type strain *S. erythraea* NRRL 23338 and a high-producing industrial stain S0, and their coding genes were overexpressed via φC31 integration driven by the two strongest promoter variants *p*_*SACE_2101_s32*_ and *p*_ermE*_23_ screened from different libraries. Combining promoter engineering and gene overexpression, erythromycin production was improved by as high as 137.24-folds in different recombinant strains, confirming that EryCIV, EryBVI, EryCVI, EryBV, EryBIV, and EryBIII, which are involved in monosaccharide synthesis and addition, are the key limiting factors in erythromycin biosynthesis in *S. erythraea* NRRL 23338. Our work enriches the accessible genetic toolkit in the important erythromycin-producing host *S. erythraea* NRRL 23338, and also sheds light on promoter engineering and expression fine-tuning in the similar filamentous actinomycete hosts.

## Materials and Methods

### Strains, Culture Conditions and Materials

Strains used in this study were listed in the Supplementary Table S5. *Saccharopolyspora erythraea* NRRL 23338 was the parental strain with low erythromycin production (Accession number: NC_009,142) (Oliynyk et al., 2007). *S. erythraea* S0 was an industrial strain with high erythromycin production in laboratory stock. *E. coli* DB3.1 was the recipient strain for constructing *ccdB*-containing plasmids. *E. coli* DH5α was the host strain for other plasmid construction. *E. coli* strains were cultivated in LB medium. *S. erythraea* strains were cultivated in R2YE liquid medium or on R2YE agar plates (Kieser et al., 2000). MS agar medium was used for conjugational transfer (Kieser et al., 2000). Antibiotics were added as follows when it was necessary: apramycin, 50 μg/ml for both *S. erythraea* and *E. coli*; hygromycin, 80 μg/ml for *S. erythraea* and 200 μg/ml for *E. coli*.; kanamycin and chloramphenicol, 25 μg/ml each for *E. coli* ET12567/pUZ8002; and nalidixic acid, 25 μg/ml for *S. erythraea* isolation after conjugational transfer.

### Construction of eGFP Expression Plasmids and *S. erythraea* Strains

The primers used in this study were listed in Supplementary Table S6. Potential promoter regions were first scanned by the PromoterHunter tool of phiSITE (http://www.phisite.org/) (Klucar et al., 2010), and 23 predicted endogenous promoters before housekeeping genes were amplified form *S. erythraea* NRRL 23338 genome using the corresponding primers. Then these promoter fragments were fused with the e*gfp* gene and integrated with the pSET152-hyg backbone by *in-vitro* homologous recombination (ClonExpress MultiS One Step Cloning Kit, Vazyme, China), respectively, generating 23 integrative eGFP expression plasmids (Supplementary Table S5).

Then these 23 eGFP expression plasmids, as well as two laboratory-stocked plasmids pSET152-hyg-ermE*p-egfp (ATG) and pSET152-hyg-*rpsL(XC)p*-egfp (ATG) (Tu et al., 2021), were transformed into *S. erythraea* NRRL 23338 by conjugational transfer according to the general protocols (Kieser et al., 2000), generating a series of eGFP expression strains (Supplementary Table S5).

### Fluorescence Detection and Analysis

The fluorescence intensities of eGFP expression strains were detected at Ex 488 nm/Em 520 nm using a microplate reader (Bio Tek, Neo2) or a fluorescence microscope (Leica DM5000B). To evaluate promoter strengths, each *S. erythraea* eGFP expression strain was first cultivated in 2-ml R2YE liquid medium in a 24-well plate at 32°C and 250 rpm for 36 h, after which 200-μL seed broth was transferred into 2-ml R2YE liquid medium for another 28 h-cultivation at 32°C and 250 rpm. Then the mycelium culture was used for fluorescence microscopy and analysis on microplate reader. In fluorescence microscopy, the mycelium was observed under the ×40 objective lens, and the fluorescent image of mycelium was taken at an exposure time of 200 m. For microplate reader detection, 200-μL mycelium culture was added into a 96-well assay plate (Corning Incorporated, United States ) to determine fluorescence signal and biomass. Finally, the relative fluorescence of each sample was normalized by dividing its fluorescence value by the corresponding biomass value. For each sample, three biological replicates were analyzed.

### Construction of Promoter Libraries

Promoter libraries were constructed using Golden-Gate strategy (Bao et al., 2018). Starting from plasmids pSET152-hyg-*p*_*SACE_2101*_-egfp (ATG) and pSET152-hyg-ermE*p-egfp (ATG) harboring *p*_*SACE_2101*_ and *p*_ermE*_, respectively, the *ccdB* counter-selection marker was used to replace the varied regions of each plasmid. Specifically, for *p*_*SACE_2101*_, the 28-bp region containing the -10 box, spacer sequences, and the -35 box was replaced by the *ccdB* expression cassette by *in-vitro* homologous recombination. While for *p*_ermE*_, the 18-bp spacer region was replaced by the *ccdB* expression cassette in a similar way. In this way, two *ccdB*-containing helper plasmids, pSET152-hyg-*p*_*SACE_2101*_-egfp (ATG)-ccdB and pSET152-hyg*-p*_ermE*_-egfp (ATG)-ccdB were obtained.

The double-stranded DNA (dsDNA) fragments containing the varied regions and *Bsa* I cohesive ends were generated by annealing of degenerate primers at 95°C for 2 min. Then the dsDNA and the corresponding *ccdB*-containing helper plasmid were integrated by Golden-Gate assembly and transferred to *E. coli* DH5α competent cells to construct two libraries of plasmid pSET152-hyg-*p*_*SACE_2101*_–1035 (lib)-egfp (ATG) and pSET152-hyg-*p*_ermE*_-spacer (lib)-egfp (ATG). For each library, around 10,000 colonies were collected for plasmid extraction. The extracted plasmids were first transformed into *E. coli* ET12567/pUZ8002, generating ET/pSET152-hyg-*p*_*SACE_2101*_–1035 (lib)-egfp (ATG) and ET/pSET152-hyg-*p*_ermE*_-spacer (lib)-egfp (ATG) (around 22,000 ET colonies were collected for each library), and then transformed into *S. erythraea* NRRL 23338 by conjugational transfer to generate *S. erythraea/*pSET152-hyg-*p*_*SACE_2101*_–1035 (lib)-egfp (ATG) (abbreviated to SACE_2101 (lib)) and *S. erythraea/*pSET152-hyg-*p*_ermE*_-spacer (lib)-egfp (ATG) (abbreviated to ermE*(lib)), respectively. To ensure the mutation efficiency, for each library, ten emerging colonies after conjugational transfer were randomly picked and cultivated in 2-ml antibiotic-containing R2YE liquid medium at 32°C and 250 rpm for 3 days. The genomes were extracted from the mycelia (BIOMIGA bacterial gDNA isolation kit, China) and used as the templates to amplify the varied regions in promoters by PCR. The PCR products were subjected to Sanger sequencing to ensure that all picked transformants represented different genotypes from each other. At last, for each *S. erythraea* library, around 50,000 transformants were collected for further promoter screening.

### Droplet Microfluidic Based Sorting for Promoter Screening

*S. erythraea* strains were cultivated on R2YE agar plates at 34 °C for 7 days for sporulation. The spores were suspended and washed by sterile water, and were re-suspended in 5-ml R2YE liquid medium that was filtered by 0.22-μm membrane, and the spore suspension were filtered through eight-layered sterilized lens paper for two or three times to remove mycelium. 10-μL spore suspension was applied on the hemocytometer to determine the spore concentration by microscopy observation under the ×20 objective lens (Leica DM5000B).

To generate droplets, spore suspension whose final concentration was 2×10^6^ spores/mL was used as the aqueous phase, while HFE-7500 fluorinated fluid (3 M, United States ) with 1.0% (w/w) surfactant (RAN Biotechnologies, United States ) was used as the oil phase. The aqueous and oil phases were pumped into the microfluidic droplet-generating device (Tu et al., 2021) at flow rates of 400 μL/h and 800 μL/h, respectively, generating droplets whose diameter were around 90 nm. The generated droplets were collected in a 1.5-ml tube for microscopy observation or in a 1-ml syringe for further FADS sorting.

The collected droplets were incubated at 34°C for 3 days. During cultivation, the mycelium-containing droplets were observed by fluorescent microscope under the ×20 objective lens at different time intervals of day 1, day 2, and day 3 to choose the optimal sorting time. For sorting, droplets in the 1-ml syringe were pumped into the microfluidic droplet sorting device (Tu et al., 2021) at a flow rate of 20 μL/h. At the same time, the spacing oil HFE-7500 was pumped at a flow rate of 1000 μL/h to separate droplets. Excited by the 488-nm laser, the emitted 520-nm fluorescence signal of droplets was detected by the photomultiplier tube (PMT), and different PMT values of droplets reflected their different fluorescence intensities. The droplets with desired PMT values were forced to deflect into the sorting channel by 700-V voltage at an average frequency of 15 Hz. The sorted droplets were spread on and cultivated on R2YE agar plates at 34°C for 4 to 5 days until colonies emerged. Then the colonies were picked and transferred into R2YE liquid medium for mycelium cultivation at 32°C and 250 rpm, and the fluorescence intensities of sorted strains were verified as described in “Fluorescence detection and analysis”. The varied region in promoter of each sorted strain was amplified by PCR using the genome template extracted from the mycelium, and was subjected to Sanger sequencing to obtain the correspondence of fluorescence intensity and promoter sequence.

eGFP expression plasmids harboring all initially sorted promoter variants *p*_*SACE_2101_s32*_, *p*_*SACE_2101_s33*_, *p*_*SACE_2101_s43*_, *p*_*SACE_2101_s35*_, *p*_ermE*_s23_, *p*_ermE*_s41_, *p*_ermE*_s42_, and *p*_ermE*_s43_, were reconstructed by *in-vitro* homologous recombination as described in “Construction of eGFP expression plasmids and *S. erythraea* strains”, generating plasmids pSET152-hyg-*p*_*SACE_2101_s32*_-egfp (ATG), pSET152-hyg-*p*_*SACE_2101_s33*_-egfp (ATG), pSET152-hyg-*p*_*SACE_2101_s43*_-egfp (ATG), pSET152-hyg-*p*_*SACE_2101_s35*_-egfp (ATG), pSET152-hyg-*p*_ermE*_s23_-egfp (ATG), pSET152-hyg-*p*_ermE*_s41_-egfp (ATG), pSET152-hyg-*p*_ermE*_s42_-egfp (ATG), and pSET152-hyg-*p*_ermE*_s43_-egfp (ATG). These plasmids were transferred into the blank strain *S. erythraea* NRRL23338, resulting in eight eGFP expression *S. erythraea* strains *S. erythraea*/pSET152-hyg-*p*_*SACE_2101_s32*_-egfp (ATG), *S. erythraea*/pSET152-hyg-*p*_*SACE_2101_s33*_-egfp (ATG), *S. erythraea*/pSET152-hyg-*p*_*SACE_2101_s43*_-egfp (ATG), *S. erythraea*/pSET152-hyg-*p*_*SACE_2101_s35*_-egfp (ATG), *S. erythraea*/pSET152-hyg-*p*_ermE*_s23_-egfp (ATG), *S. erythraea*/pSET152-hyg-*p*_ermE*_s41_-egfp (ATG), *S. erythraea*/pSET152-hyg-*p*_ermE*_s42_-egfp (ATG), and *S. erythraea*/pSET152-hyg-*p*_ermE*_s43_-egfp (ATG). The fluorescence intensities of these re-constructed strains were further determined in a similar way.

### Real-Time Quantitative PCR Analysis

*S. erythraea* NRRL 23338 and S0 were cultivated in R2YE liquid medium at 32°C and 220 rpm, and 1-ml broth after 3, 5 and 7 days of cultivation of each strain was used for RNA isolation according to the manufacturer protocols (TIANGEN RNAprep pure cell/bacteria kit, China), respectively. After isolation, RNA quality was assessed by gel electrophoresis, and the concentration of each sample was determined by the Nanodrop spectrophotometer (NanoDrop Technologies, United States ). The extracted RNA was used as the template to synthesize cDNA using the reverse transcription kit (ReverTra Ace qPCR RT Master Mix with gDNA Remover, TOYOBO, Japan). The cDNA was used as the template in the following real-time quantitative PCR (TaKaRa SYBRGREEN real time PCR Mix, Japan) in the thermal cycler (LightCycler 480II, Roche, Switzerland). Each gene was amplified using a specific primer pair, and the housekeeping gene *sigA* (*SACE_1801*) was employed as the internal reference (Supplementary Table S6). Gene relative expression levels were calculated using the comparative cycle threshold method (Livak and Schmittgen, 2001).

### Construction of *Ery* Gene Overexpression *S. erythraea* Strains

Each *ery* gene for overexpression was amplified from *S. erythraea* NRRL 23338 genome by PCR using the corresponding primers. The promoter variants *p*_*SACE_2101_s32*_ and *p*_ermE*_s23_ were amplified by PCR using the re-constructed plasmids pSET152-hyg-*p*_*SACE_2101_s32*_-egfp (ATG) and pSET152-hyg-*p*_ermE*_s23_-egfp (ATG) as the templates. Then each promoter fragment, *ery* gene fragment and pSET152-hyg backbone were assembled by *in-vitro* homologous recombination to construct a series of integrative overexpression plasmids pSET152-hyg-*p*_*SACE_2101_s32*_-SACE_0716, pSET152-hyg-*p*_*SACE_2101_s32*_-SACE_0717, pSET152-hyg-*p*_*SACE_2101_s32*_-SACE_0718, pSET152-hyg-*p*_*SACE_2101_s32*_-SACE_0719, pSET152-hyg-*p*_*SACE_2101_s32*_-SACE_0720, pSET152-hyg-*p*_*SACE_2101_s32*_-SACE_0731, pSET152-hyg-*p*_ermE*_s23_-SACE_0716, pSET152-hyg-*p*_ermE*_s23_-SACE_0717, pSET152-hyg-*p*_ermE*_s23_-SACE_0718, pSET152-hyg-*p*_ermE*_s23_-SACE_0719, pSET152-hyg-*p*_ermE*_s23_-SACE_0720, pSET152-hyg-*p*_ermE*_s23_-SACE_0731. Then these plasmids were transformed into *S. erythraea* NRRL 23338 by conjugational transfer, generating different overexpression strains *S. erythraea*/pSET152-hyg-*p*_*SACE_2101_s32*_-SACE_0716, *S. erythraea*/pSET152-hyg-*p*_*SACE_2101_s32*_-SACE_0717, *S. erythraea*/pSET152-hyg-*p*_*SACE_2101_s32*_-SACE_0718, *S. erythraea*/pSET152-hyg-*p*_*SACE_2101_s32*_-SACE_0719, *S. erythraea*/pSET152-hyg-*p*_*SACE_2101_s32*_-SACE_0720, *S. erythraea*/pSET152-hyg-*p*_*SACE_2101_s32*_-SACE_0731, *S. erythraea*/pSET152-hyg-*p*_ermE*_s23_-SACE_0716, *S. erythraea*/pSET152-hyg-*p*_ermE*_s23_-SACE_0717, *S. erythraea*/pSET152-hyg-*p*_ermE*_s23_-SACE_0718, *S. erythraea*/pSET152-hyg-*p*_ermE*_s23_-SACE_0719, *S. erythraea*/pSET152-hyg-*p*_ermE*_s23_-SACE_0720, *S. erythraea*/pSET152-hyg-*p*_ermE*_s23_-SACE_0731 (Supplementary Table S5).

### Fermentation and Erythromycin Production Analysis

To evaluate erythromycin production, each *S. erythraea* strain was first cultivated in 3 ml seed medium in a 24-well plate (BIO-YD, China) under 32°C and 250 rpm. And then, 300 μL seed broth was transferred into 3-ml fermentation medium in a 24-well plate for 7 days cultivation under 32°C and 250 rpm. The composition of seed medium was as follows: glucose, 10 g/L; tryptone, 4 g/L; yeast extract, 4 g/L; MgSO_4_, 0.5 g/L; KH_2_PO_4_, 2.0 g/L; K_2_HPO_4_, 4 g/L. The composition of fermentation medium was as follows: starch, 20 g/L; dextrin, 20 g/L; soybean powder, 15 g/L (NH_4_)_2_SO_4_, 4 g/L; CaCO_3_, 6 g/L; soybean oil, 5 ml/L. For erythromycin production analysis, the harvested fermentation broth was extracted by ethyl acetate for two times, and the organic phase was collected and dried in vacuo. The residue was dissolved in 300-μL acetonitrile and filtered by 0.22 μm membrane to prepare the HPLC sample. Erythromycin production was evaluated using HPLC method (Agilent 1260 Infinity II, United States ) at 30°C with the C_18_ column (4.6 × 150 mm, 4 μm) (InfinityLab Poroshell 120 EC-C18, Agilent, United States ). The mobile phases were 55% of solvent A (KH_2_PO_4_ solution) and 45% of solvent B (acetonitrile) at a flow rate of 0.8 ml/min. UV signals were detected at 215 nm.

## Results

### Characterization of Native Promoters in *S. erythraea* NRRL 23338

**FIGURE 1:**
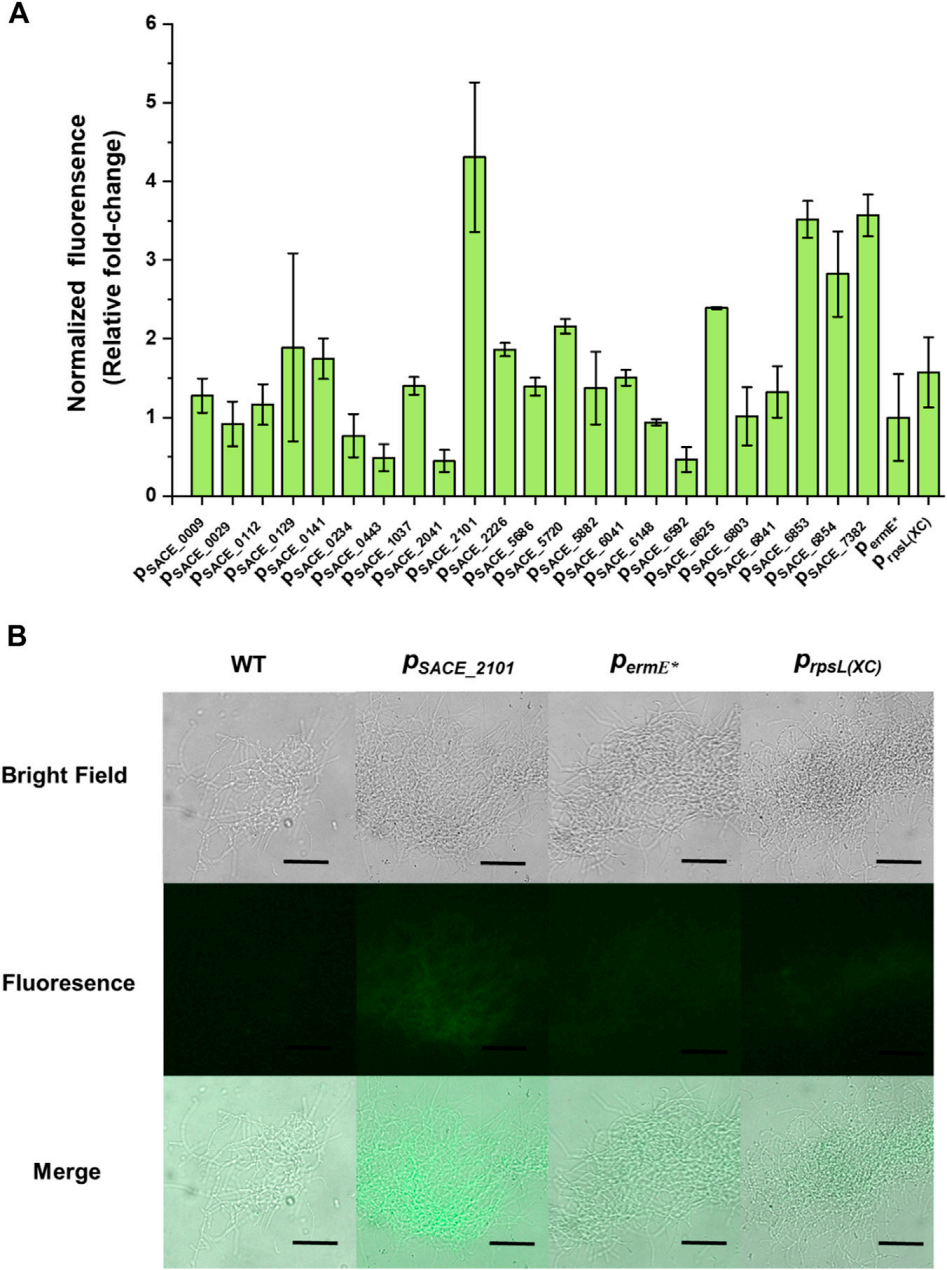
Native promoters characterization in *S. erythraea* NRRL 23338. **(A)** Relative normalized green fluorescence of different eGFP-expressing strains compared to the *p*_ermE*_-harboring strain (fold-change). Error bars represented the standard deviation of three parallel samples. **(B)** Fluorescence microscope observation of wild-type strain *S. erythraea* NRRL 23338 and eGFP-expressing strains harboring promoters *p*_*SACE_2101*_, *p*_ermE*_, and *p*_*rpsL(XC)*_. Scale bar: 50 μm.

Promoters are key regulatory elements in comprehensive metabolic engineering. For gene expression in most *Streptomyces* as well as *S. erythraea*, the most widely used promoters are *p*_*ermE*_ and its variant *p*_ermE*_ (Bibb et al., 1985). To provide more accessible promoters for fine-tuning gene expression in *S. erythraea*, we first selected and characterized 23 native promoters upstream of housekeeping genes in *S. erythraea* NRRL 23338. These housekeeping genes widely spread across *S. erythraea* genome and are involved in a series of important primary processes including gene transcription, glycolysis, translational elongation, and amino-acyl tRNA synthesis (Supplementary Table S1). The strengths of these promoter candidates were evaluated using the enhanced green fluorescent protein (eGFP) as the reporter. Besides, two heterologous promoters, *p*_ermE*_, which is commonly regarded as a strong promoter in *Streptomyces* (Olano et al., 2014; Ji et al., 2018; Zhang et al., 2021), and *p*_*rpsL(XC)*_ derived from *Xylanimonas cellulosilytica*, which was characterized as a strong promoter in *S. lividans* in previous works (Shao et al., 2013; Cobb et al., 2015), were also cloned to drive eGFP expression as controls. The normalized green fluorescence strength of strains harboring different promoters were calculated and compared to that of the *p*_ermE*_-carrying strain to reflect their relative promoter strengths. The result showed that six native promoters, *p*_*SACE_2101*_, *p*_*SACE_5720*_, *p*_*SACE_6625*_, *p*_*SACE_6853*_, *p*_*SACE_6854*_, and *p*_*SACE_7382*_, were significantly stronger than *p*_ermE*_, among which, *p*_*SACE_2101*_, *p*_*SACE_6853*_, and *p*_*SACE_7382*_, exhibited 4.31, 3.52 and 3.57 folds of strengths compared to *p*_ermE*_, respectively (Figures 1A, B). For the heterologous promoters, *p*_ermE*_ and *p*_*rpsL(XC)*_ showed no significantly different activity in *S. erythraea* NRRL 23338, and both of them were much weaker than many of the native promoters (Figures 1A, B). In contrast, *p*_*rpsL(XC)*_ exhibited more than 10-fold higher activity than *p*_ermE*_ in *S. lividans* (Shao et al., 2013). These results indicated that promoter activities may vary significantly among different expression hosts, and thus precise promoter characterization and engineering in the target expression host is necessary.

### Promoter Engineering and Screening Using Droplet-Based Microfluidic Platform

**FIGURE 2:**
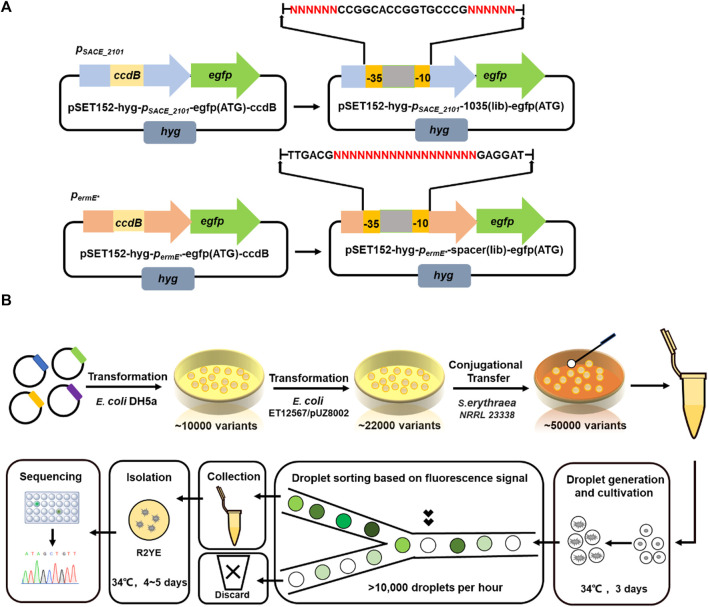
Promoter engineering and screening using droplet-based microfluidic platform. **(A)** Schematic diagrams of two plasmid libraries pSET152-*p*_*SACE_2101*_–1035 (lib)-egfp (ATG) and pSET152-*p*_ermE*_-spacer (lib)-egfp (ATG). “NNN” indicated the varied regions. **(B)** Work flow of promoter library construction and droplet microfluidic based high-throughput screening.

To facilitate gene expression fine-tuning in *S. erythraea* NRRL 23338, we intended to characterize a set of promoters with different strengths through promoter engineering. In the previous study, we have established a droplet-based microfluidic platform that can facilitate rapid promoter engineering and screening in *Streptomyces*, and we successfully obtained different *p*_ermE*_ variants by mutating its 18-bp spacer sequences between -10 and -35 regions (Tu et al., 2021). Considering that both the -10 and -35 core regions and the spacer sequences can influence promoter strength, we decided to construct promoter libraries in two ways. Starting from the heterologous promoter *p*_ermE*_ and the strongest native promoter *p*_*SACE_2101*_, we followed the previous method of random mutagenesis in 18-nt spacer sequences to engineer *p*_ermE*_, and applied the other method of mutating the -10 and -35 regions to engineer *p*_*SACE_2101*_, resulting in two plasmid libraries pSET152-*p*_*SACE_2101*_–1035 (lib)-egfp (ATG) and pSET152-*p*_ermE*_-spacer (lib)-egfp (ATG) carrying promoter variants (Figure 2A). Approximately 10,000 *E. coli* DH5α transformants of each library were collected in plasmid construction. The plasmids were transformed into *S. erythraea* NRRL 23338 by conjugational transfer, and two *S. erythraea* libraries containing around 50,000 transformants, each integrated with pSET152-*p*_*SACE_2101*_–1035 (lib)-egfp (ATG) or pSET152-*p*_ermE*_-spacer (lib)-egfp (ATG), were obtained respectively, named SACE_2101 (lib) and ermE*(lib) (Figure 2B). In this process, several transformants of each library were randomly picked for Sanger sequencing to evaluate the efficiency of library construction, and all sequenced variants represented different sequences in their varied regions (Supplementary Figure S1).

**FIGURE 3:**
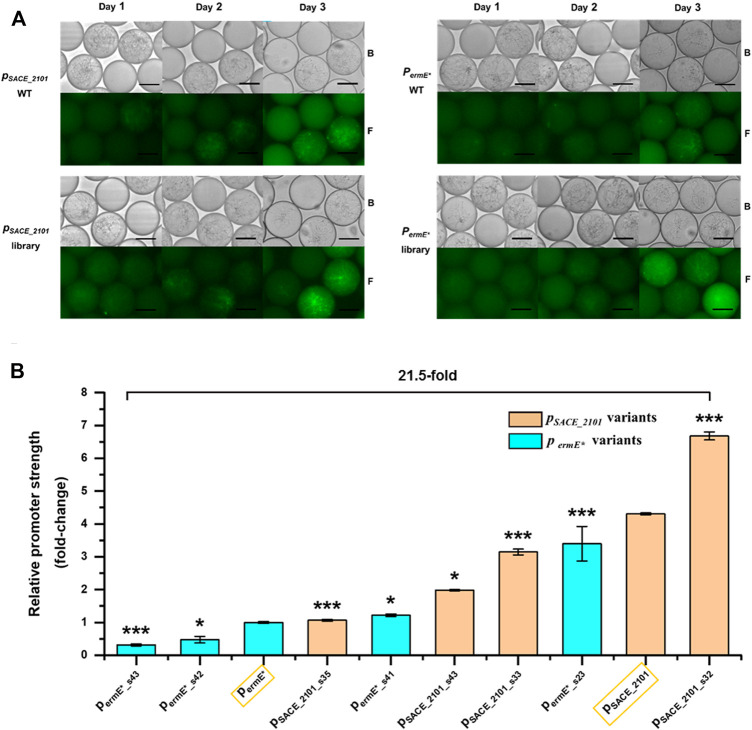
Promoter screening by FADS. **(A)** Fluorescence microscopy of droplets containing wild-type stains (*p*_*SACE_2101*_ WT, *p*_ermE*_ WT) and variants (*p*_*SACE_2101*_ library, *p*_ermE*_ library) at different time intervals. B: Bright field. F: Fluorescence. Scale bar: 50 μm. **(B)** Relative strengths of the screened promoter variants in libraries SACE_2101 (lib) (*p*_*SACE_2101*_ library) and ermE*(lib) (*p*_ermE*_ library) compared to their corresponding WTs (Surrounded by yellow frames). Error bars represented the standard deviation of three biological samples. *p ≤ 0.05 and ***p ≤ 0.001 (Student’s two-tailed *t*-test).

Then, spores of SACE_2101 (lib) and ermE*(lib), as well as their wild-types *S. erythraea*/pSET152-*p*_*SACE_2101*_-egfp (ATG) and *S. erythraea*/pSET152-*p*_ermE*_-egfp (ATG), were collected and encapsulated in droplets for cultivation, respectively. And the fluorescence of each sample was observed at different time intervals. We found that *S. erythraea* spores can germinate normally in droplet environment and the droplets were fully filled after 1-day cultivation (Figure 3A). *S. erythraea*/pSET152-*p*_*SACE_2101*_-egfp (ATG) and SACE_2101 (lib) began to exhibit green fluorescence at day 1, and the fluorescence intensity continued to enhance within 3 days. While *S. erythraea*/pSET152-*p*_ermE*_-egfp (ATG) and ermE*(lib) did not exhibit obvious green fluorescence until the cultivation time was prolonged to 3 days (Figure 3A). Thus, we chose day 3 as the sorting time point in the following FADS screening.

To screen out promoters of various strengths, PMT thresholds were adjusted to designate different gates in FADS screening based on the green fluorescence intensities of droplets in each library, so that promoter variants of desired strengths can be sorted purposefully. The sorted droplets were spread on agar plates for variant isolation and Sanger sequencing (Figure 2B). To avoid effects of random genome mutations, strains harboring each promoter variant were re-constructed and analyzed. In the library SACE_2101 (lib) derived from the native promoter *p*_*SACE_2101*_, four promoter variants *p*_*SACE_2101_s32*_, *p*_*SACE_2101_s33*_, *p*_*SACE_2101_s43*_ and *p*_*SACE_2101_s35*_ were finally screened out representing 155, 73, 30 and 25% of strength, compared to the wild-type *p*_*SACE_2101*_ (Figure 3B). In the library ermE*(lib) derived from the heterologous promoter *p*_ermE*_, four promoter variants *p*_ermE*_s23_, *p*_ermE*_s41_, *p*_ermE*_s42_, *p*_ermE*_s43_ were finally screened out exhibiting 340, 122, 47 and 31% of strength, compared to the original *p*_ermE*_ (Figure 3B). The mutated regions of these sorted variants were sequenced and listed in Supplementary Table S2. All promoter variants were arranged according to their relative strength, where the weakest and the strongest ones represented 21.5-fold variation (Figure 3B).

### Comparative qRT-PCR Analysis of *ery* Genes in *S. erythraea* NRRL 23338 and S0

**FIGURE 4:**
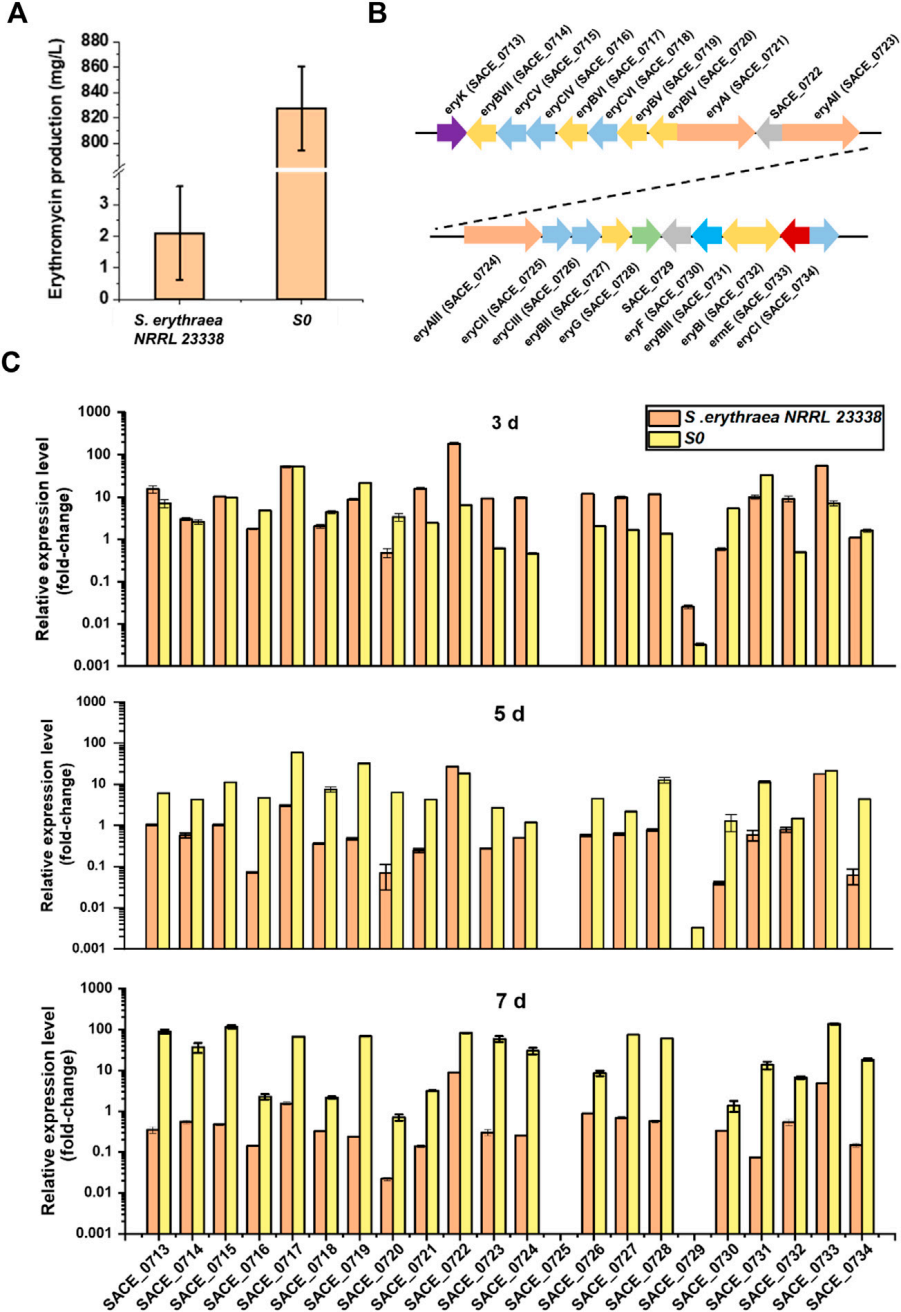
Comparative qRT-PCR analysis of *ery* genes in the low-producing strain *S. erythraea* NRRL 23338 and the high-producing strain S0. **(A)** Erythromycin productions of *S. erythraea* NRRL 23338 and S0 after 7-days cultivation in 24-well-plate. **(B)** Schematic representation of erythromycin biosynthetic gene cluster (*ery*). **(C)** Relative expression levels of *ery* genes in *S. erythraea* NRRL 23338 and S0 at day 3, day 5 and day 7. The vertical axis was the logarithmic axis. Error bars represented the standard deviation of three experiments.

Comparing transcriptional levels of targeted genes in different strains are usually employed to determine key under-expressed genes for constructing overproducing strains for improved yields (Bu et al., 2021). To identify the potential limiting factors that restricted erythromycin biosynthesis in the wild-type strain, we performed comparative qRT-PCR analysis of the low-producing strain *S. erythraea* NRRL 23338 and a high-producing industrial strain S0 in our laboratory stock, which represented over 400-fold variation in erythromycin titers in 24-well-plate cultivation (Figure 4A). The expression levels of twenty-two *ery* genes in these two strains were assessed using the mycelium harvested after 3, 5, and 7 days of cultivation, respectively, while the housekeeping gene *sigA* (*SACE_1801*, encoding an RNA polymerase sigma factor) was used as the internal reference gene (Figure 4B). Generally speaking, almost all *ery* genes in *S. erythraea* NRRL 23338 showed low expression levels compared to those in S0, especially at the late fermentation stage (day 5 and day 7) (Figure 4C). In S0, *ery* gene expression kept on elevating through the whole fermentation process, especially during day 5 to day 7, when secondary metabolism was active and erythromycin was accumulated rapidly. In contrast, *ery* gene expression in *S. erythraea* NRRL 23338 significantly decreased through fermentation, where the expression levels in the late stage (day 5 and day 7) was obviously much lower than those in the early stage (day 3) (Figure 4C).

Improving key gene expressions levels in BGCs usually contributes to higher productions of target secondary metabolites. For example, the transcriptome analysis revealed that the insufficient transcription of *dpt* genes was probably the major factor contributing to the low daptomycin production in the native producer *S. roseosporus* NRRL 11379, and improving gene expression by promoter engineering resulted in significant improvement in lipopeptide production (Ji et al., 2022). We speculated that the consistency of gene expression in the late fermentation stage was the key factor for the enhanced erythromycin production in the high-producing strain S0, while the insufficient transcription of *ery* genes would lead to low yield in the wild-type host. Generally speaking, enzyme expression is prior to product accumulation, and secondary metabolites mainly accumulate during the late fermentation stage. Thus, we focused on the relative gene transcription levels of day 5 between S0 and *S. erythraea* NRRL 23338. *ery* genes were arranged according to the fold changes of their relative transcription levels of day 5 in the two strains, and the top eight differently expressed genes were *SACE_0720*, *SACE_0734*, *SACE_0719*, *SACE_0716*, *SACE_0730*, *SACE_0718*, *SACE_0717*, and *SACE_0731* (encoding EryBIV, EryCI, EryBV, EryCIV, EryF, EryCVI, EryBVI, and EryBIII) (Figures 4B, C; Supplementary Table S3). In erythromycin biosynthesis, EryF is a P450 enzyme catalyzing hydroxylation of 6-deoxyerythronolide B into erythronolide B (Mironov et al., 2004). EryB and EryC enzymes are responsible for the biosynthesis of *l* -mycarose and *D*-desosamine from the common precursor 4-keto-6-deoxyglucose metabolized from glucose-1-P, respectively (Summers et al., 1997). The intermediate product erythronolide B will be converted to erythromycin D by successive additions of *l* -mycarose and *D*-desosamine, and then erythromycin D will be transformed to other erythromycin intermediates through a series of post modifications (Mironov et al., 2004) (Supplementary Figure S2). These low-expressing synthases in the native host were the potential key limiting factors in erythromycin biosynthesis. To confirm this hypothesis, the roles of these genes would be verified in the following experiments.

### Expression Fine-Tuning of *Ery* Genes for Improved Erythromycin Production in *S. erythraea* NRRL 23338

**FIGURE 5:**
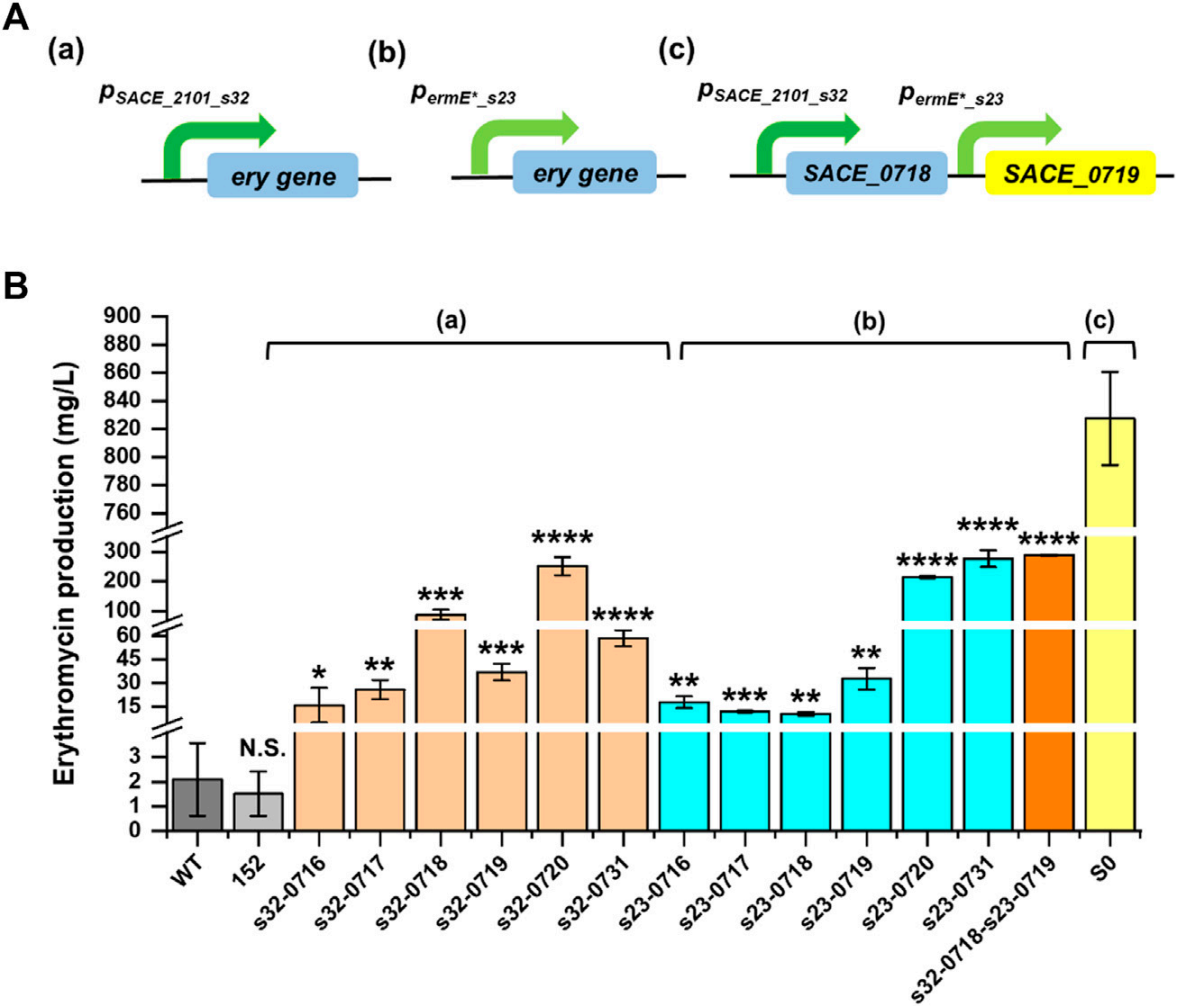
Improving erythromycin production in *S. erythraea* NRRL 23338 by promoter engineering and gene overexpression. **(A)** Constructs of single-gene overexpression driven by *p*_*SACE_2101_s32*_ (a), and *p*_ermE*_s32_ (b), and combinational overexpression of *SACE_0718* and *SACE_0719* driven by *p*_*SACE_2101_s32*_ and *p*_ermE*_s32_, respectively (c). *ery* genes: *SACE_0716*, *SACE_0717*, *SACE_0718*, *SACE_0719*, *SACE_0720*, and *SACE_0731*. **(B)** 24-well-plate fermentation results of *S. erythraea* NRRL 23338 (WT), *S. erythraea*/pSET152-hyg (152), overexpressing strains and high-producing strain S0. *p ≤ 0.05, **p ≤ 0.01, ***p ≤ 0.001, ****p ≤ 0.0001, and N.S. indicated no significant difference (Student’s two-tailed *t*-test).

Overexpressing the rate-limiting genes is usually a direct and effective way to improve the secondary biosynthesis and metabolite production (Bu et al., 2021). In order to enhance erythromycin biosynthesis, we intended to combine promoter engineering and gene overexpression strategy to enhance the expression of the potential eight key limiting enzymes in *S. erythraea* NRRL 23338. At the very beginning, a preliminary experiment was conducted that three genes, *SACE_0720*, *SACE_0734*, and *SACE_0730* were selected to be overexpressed driven by the weak promoter *p*_*rpsL(XC)*_. However, for some unknown reason, *SACE_0734* and *SACE_0730* overexpressing strains did not produced erythromycin any more. While *SACE_0720* overexpression contributed to an erythromycin titer of 185.44 mg/L, representing 88.3 folds compared to that of the WT strain (Supplementary Figure S3; Supplementary Figure S4). Given these results, *SACE_0734* and *SACE_0730* were no longer to be overexpressed using the engineered promoters in the following experiments. Next, two strongest promoter variants screened from different libraries, *p*_*SACE_2101_s32*_ and *p*_ermE*_s23_, were employed to drive the expression of the rest six genes of *SACE_0716*, *SACE_0717*, *SACE_0718*, *SACE_0719*, *SACE_0720*, and *SACE_0731*, respectively (Figure 5A). The resultant two series of pSET152-derived overexpression plasmids were integrated onto *S. erythraea* NRRL 23338 genome via φC31 integration. At the same time, a strain integrated with a blank plasmid pSET152-hyg was also constructed and used as the control in the fermentation. The results showed that the blank-plasmid-harboring strain and the wild-type strain *S. erythraea* NRRL 23338 produced similar amounts of erythromycin, and all single-gene overexpression strains represented significantly enhanced production compared to the wild-type strain (Figure 5B). This was consistent with the transcription analysis result and verified the key limiting roles of these six enzymes in erythromycin biosynthesis in the wild-type strain *S. erythraea* NRRL 23338. The most outstanding single-gene overexpressing strain *S. erythraea/*pSET-hyg-*p*_ermE*_s23_-*SACE_0731* represented as high as 275.83 mg/L (131.42 folds of improvement) of erythromycin in 24-well-plate fermentation (Figure 5B; Supplementary Figure S3; Supplementary Table S4). For the overexpression of genes *SACE_0717*, *SACE_0718*, *SACE_0719*, and *SACE_0720*, the *p*_*SACE_2101_s32*_-driven strains performed better than the other series, and the titers of recombinant strains were improved by 12.31 to 119.66 folds compared to the wild-type strain. Interestingly, we found that the stronger promoter did not always mean the higher production. We have characterized that *p*_*SACE_2101_s32*_ was 2-fold stronger than *p*_ermE*_s32_, but for the overexpression of genes *SACE_0716* and *SACE_0731*, the recombinant strains harboring the relatively weaker promoter *p*_ermE*_s32_ led to higher productions, and the titers were enhanced by 8.50 and 131.42 folds, respectively (Figure 5B; Supplementary Table S4). To identify if this abnormal phenomenon was induced by the unexpected expression levels of the target gene, *SACE_0731* series overexpression strains were taken as examples to conduct qPCR experiments, where the weaker promoter performed dramatically better than the stronger one. The expression levels of *p*_*SACE_2101_s32*_-driven and *p*_ermE*_s23_-driven *SACE_0731* overexpression strains at day 5 were identified and compared to that of the pSET152-harboring control strain. The results showed that *p*_*SACE_2101_s32*_-*SACE_0731* and *p*_ermE*_s23_-*SACE_0731* overexpression induced 90.5 and 42.7% improvement on *SACE_0731* expression compared to the control, respectively, indicating that the stronger promoter *p*_*SACE_2101_s32*_ actually contributed to a higher expression level in *SACE_0731* overexpression (Supplementary Figure S5). However, the higher *SACE_0731* expression level did not consequently result in a higher erythromycin production. This may be due to the excess gene transcription than needed and the consequent metabolic burden for the host, or feedback inhibition of some excessive intermediates, which were harmful to efficient erythromycin biosynthesis. Thus, coordinating expression of different genes in a pathway to a proper level by using promoters of varied strengths is a better way to promote secondary metabolite production.

We also attempted to further improve erythromycin production by combinational overexpression. *SACE_0718* and *SACE_0719*, which were proved to be important to erythromycin biosynthesis, were co-overexpressed in *S. erythraea* NRRL 23338 driven by *p*_*SACE_2101_s32*_ and *p*_ermE*_s23_, respectively (Figure 5A). The fermentation result demonstrated that co-overexpression of these two genes contributed to 288.06 mg/L of erythromycin, which was 137.24 folds compared to that in the wild-type strain (Figure 5B; Supplementary Table S4). This titer was 3.27 and 8.79 folds improved compared to the single-gene overexpressing strains *S. erythraea/*pSET152-hyg-*p*_*SACE_2101_s32*_-*SACE_0718* and *S. erythraea/*pSET152-hyg-*p*_ermE*_s23_-*SACE_0719*, respectively. Collectively, by fine-tuning the expression of multiple key limiting enzymes, erythromycin production was significantly improved by 5.01 to 137.24 folds in the low-producing WT host *S. erythraea* NRRL 23338.

## Discussion

Secondary metabolism is a sophisticated process that involves a series of genes, where multiple gene expression should be coordinated to compatible levels to avoid wasting intracellular energy and metabolic flux and to maximize the yield of target products. In the WT host, the production of secondary metabolite can be limited for various reasons. The intricate regulation network and the insufficient expression of key genes may lead to inefficient secondary biosynthesis, while high intracellular accumulation of secondary metabolite may also cause feedback inhibition or even cytotoxicity which is lethal to the host cell (Wei et al., 2018; Bu et al., 2021). Promoter engineering is a commonly-used strategy to solve this problem. The inherent regulatory network in the native host that restricted secondary biosynthesis can be disrupted by replacing the native promoter with a constitutive one. On the other hand, a promoter of suitable strength can be simply used to overexpress the rate-limiting gene, transporter gene, and resistance gene to achieve higher production (Bu et al., 2021). Thus, a well-characterized promoter panel of different strengths for fine-tuning gene expression is essential for metabolic engineering. In this study, using the droplet-microfluidic based platform, we demonstrated a systematical promoter acquiring process including characterization, engineering and high-throughput screening in the rare actinomycete *S. erythraea* NRRL 23338. We successfully obtained a panel of promoters with 21.5-fold strength variation starting from the native strong promoter *p*_*SACE_2101*_ and the heterologous promoter *p*_ermE*_ out of over 100,000 variants, which is impossible for traditional plate based screening process. Since *S. erythraea* NRRL 23338 is an important erythromycin producer, these regulatory elements will enrich the genetic toolkit in this native host and can be directly used in the gene manipulation to enhance erythromycin biosynthesis by expression fine-tuning or BGC refactoring.

For secondary metabolite producers with varied yields, their transcription patterns usually represented significant differences. Several comparative-transcriptome-guided analyses have demonstrated the correlation between the expression of *ery* BGC and erythromycin production. Most *ery* genes were observed to be significantly up-regulated both in the high-producing strains Px and E3, compared to the wild-type *S. erythraea* NRRL 23338 (Peano et al., 2012; Li et al., 2013). In another classical erythromycin high producer, *ery* genes were found to consistently express in the fermentation course compared to the wild-type strain (Lum et al., 2004). Coinciding with these findings, we characterized that *SACE_0716*, *SACE_0717*, *SACE_0718*, *SACE_0719*, *SACE_0720*, and *SACE_0731*, were the most significantly different expressing genes in the late fermentation stage, indicating that their coding products EryCIV, EryBVI, EryCVI, EryBV, EryBIV, and EryBIII, are key limiting enzymes in erythromycin biosynthesis. Indeed, it is commonly believed that erythromycin production is limited by the biosynthesis and addition of glycosyl ligands (Mironov et al., 2004), which involves EryB and EryC synthases, and our results demonstrate that the low expressions of *eryB* and *eryC* coding genes may be crucial factors for the inefficient biosynthesis. However, it seems that excess gene expression is also disadvantage to maximize production. Our result demonstrated that the strongest promoter was not always the best option for gene overexpression. Thus, fine-tuning gene expression by promoter engineering is a practical way to alleviate gene expression incompatibility and promote secondary biosynthesis more smoothly.

The research paradigm demonstrated in this work can be easily applied in similar filamentous actinomycetes. However, it should be noticed that successful library construction relied on high transformation efficiency to the host, so easy access to genetic manipulation, especially efficient transformation, is the prerequisite for applying droplet-microfluidic-based promoter engineering in the non-model species. The high screening throughput of our droplet-microfluidic based platform makes it possible to rapidly acquire strong promoters as well as promoter variants with desired strengths in the target expression hosts. In the future work, with the help of CRISPR technologies, proper promoters can be knocked in to replace the native promoters upstream of multiple target genes, so that *in-situ* expression enhancement and fine-tuning can be accomplished in a scarless way (Liu et al., 2018; Liu et al., 2019). Considering that actinomycetes are the most abundant sources of natural products, our work will accelerate rational strain engineering and yield improvement of target products in actinomycete hosts.

## Data Availability

The original contributions presented in the study are included in the article/Supplementary Material, further inquiries can be directed to the corresponding authors.
