# Supplementary material for: Droplet-Microfluidic-Based Promoter Engineering and Expression Fine-Tuning for Improved Erythromycin Production in Saccharopolyspora erythraea NRRL 23338
Source: Front Bioeng Biotechnol. 2022 Apr 4;10:864977. doi: 10.3389/fbioe.2022.864977 (PMC9013967; doi:10.3389/fbioe.2022.864977)
Supplement: Supplementary file 1 [file DataSheet1.DOCX]

Supplementary Material

# Supplementary Figures and Tables

## Supplementary Figures

##
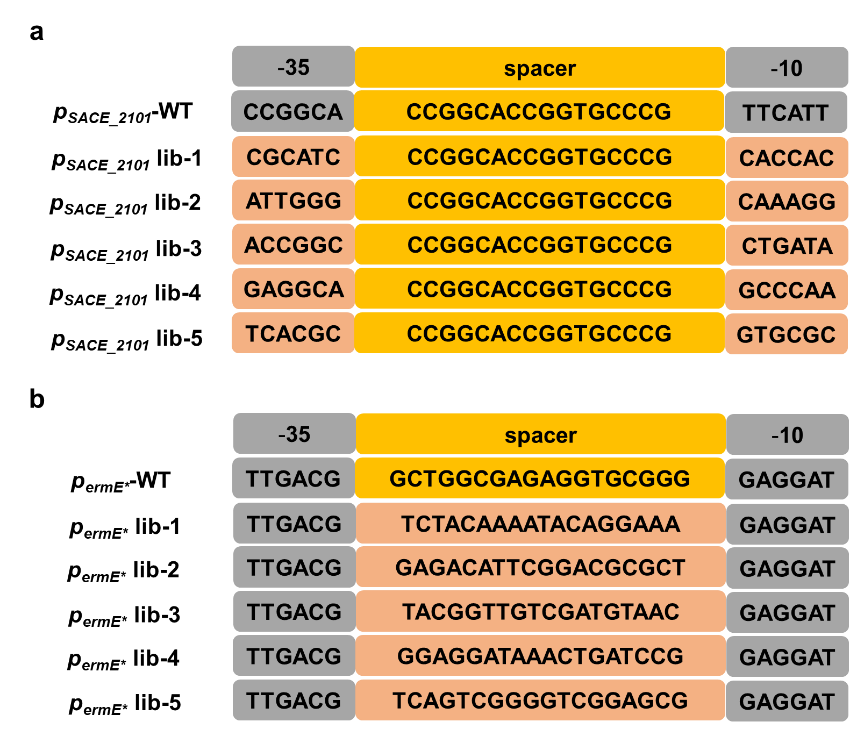


**Figure S1.** Sanger sequencing results of the randomly picked mutants in libraries SACE_2101 (lib) **(a)** and ermE*(lib) **(b)**.


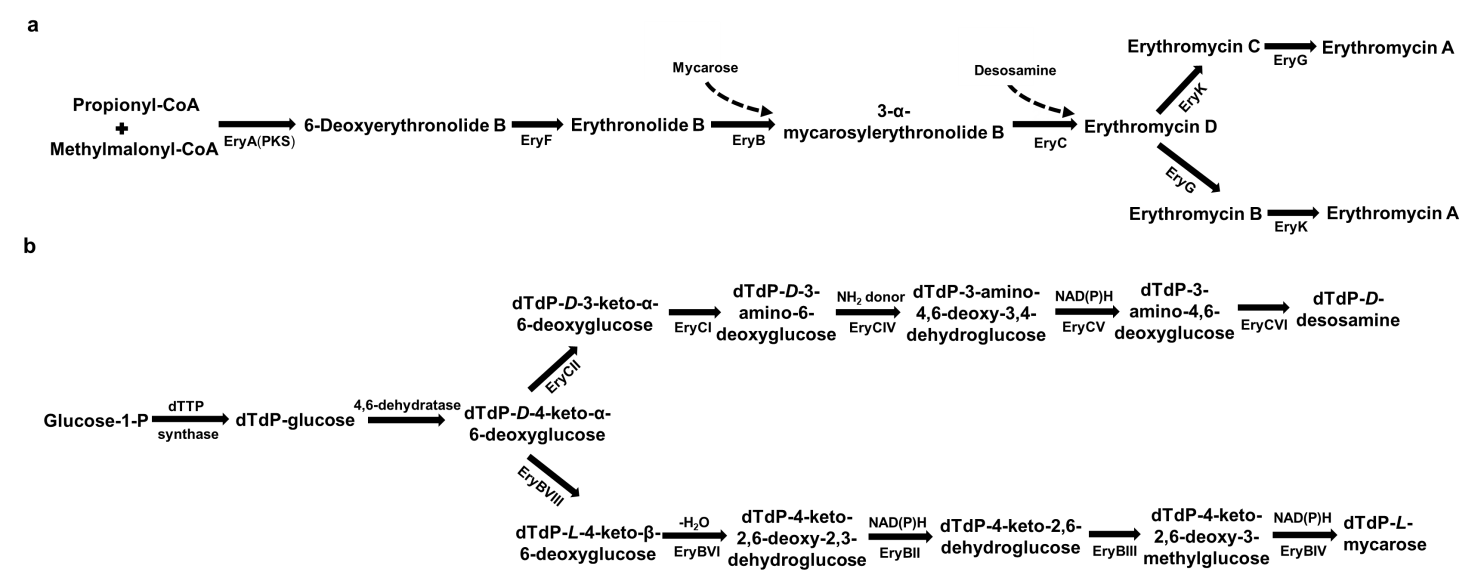


**Figure S2.** Mechanism of erythromycin biosynthesis. **(a)** Biosynthesis of erythromycin derivatives. **(b)** Biosynthesis of *L*-mycarose and *S*-desosamine.

##
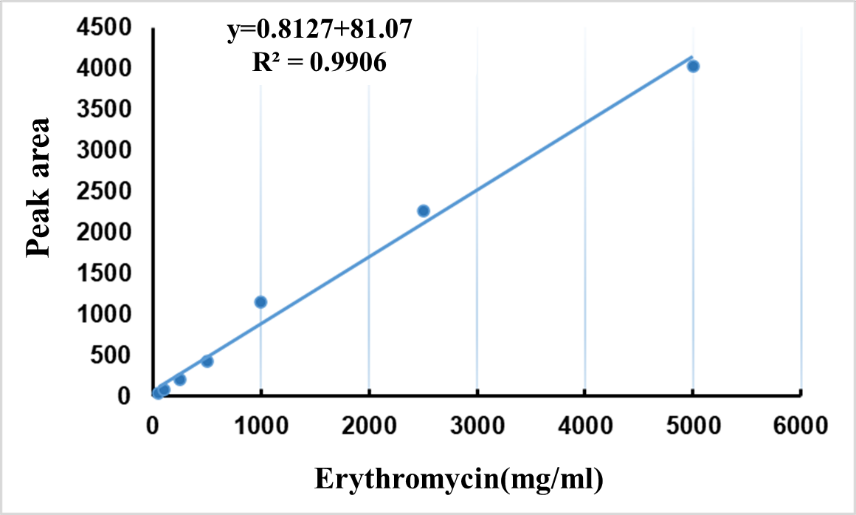


## Figure S3. Standard curve of erythromycin titers using HPLC method.


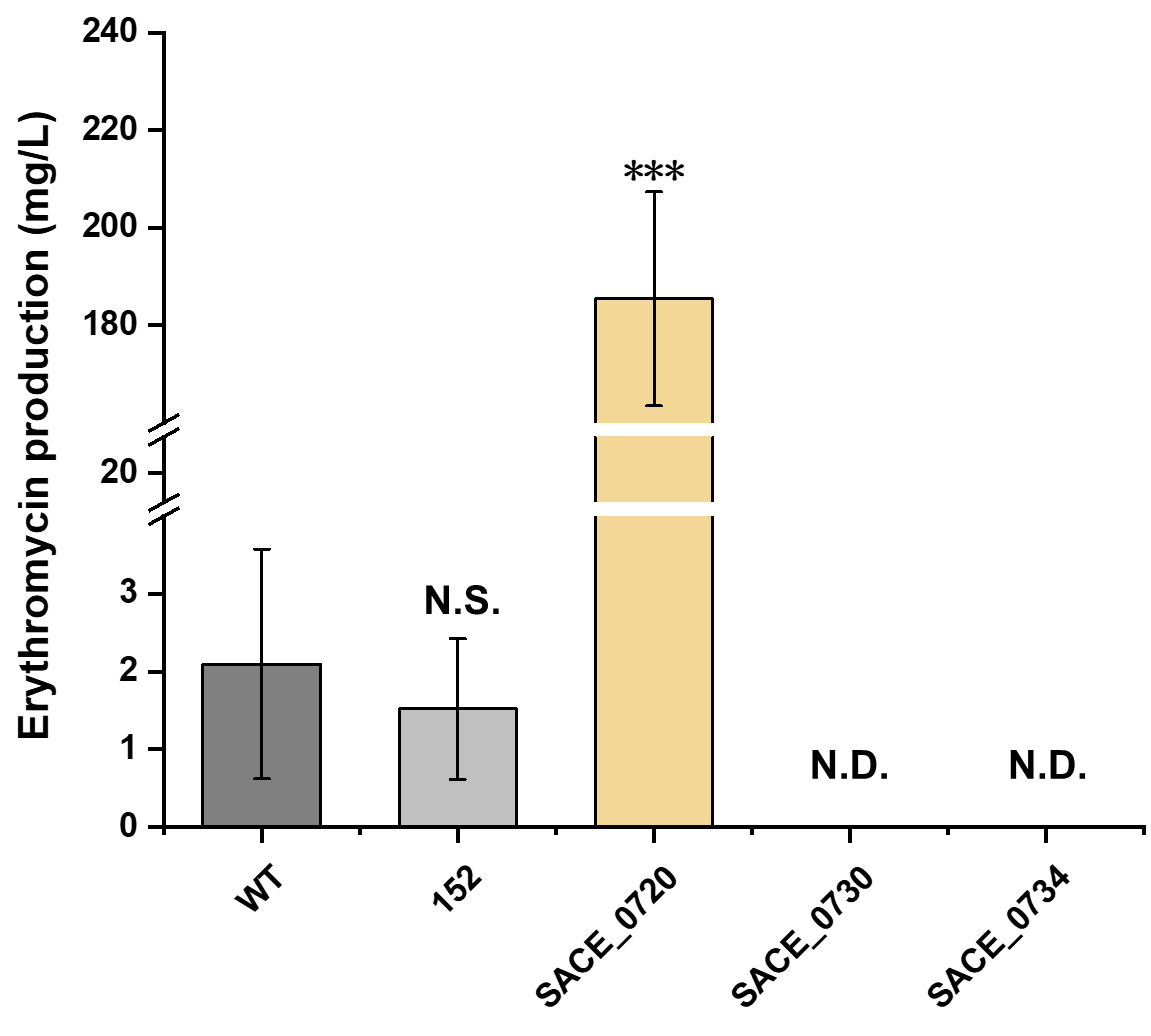


**Figure S4.** Erythromycin titers of wild-type strain *S. erythraea* NRRL 23338 (WT), pSET152-control strain *S. erythraea/*pSET152-hyg (152), and *p_rpsL(XC)_*-driven *SACE_0720*, *SACE_0730* and *SACE_0734* overexpressing strains after 7 days of 24-well-plate fermentation. Error bars represented the standard deviation of three biological samples. ****p*≤0.001, and N.S. indicated no significant difference (Student’ s two-tailed *t*-test). N.D. indicated “Not Detectable”.

**Figure S5.** Relative expression levels after 5-d cultivation of the control strain *S. erythraea*/pSET152-hyg (152), and *SACE_0731* overexpression strains *S. erythraea/*pSET152-hyg- *p_ermE*_s23_*-*SACE_0731* and *S. erythraea/*pSET152-hyg-*p_SACE_2101_s32_*-*SACE_0731* harboring *p_ermE*_s23_* and *p_SACE_2101_s32_*, respectively. The expression level of the control strain *S. erythraea*/pSET152-hyg (152) was normalized to 1. ***p*≤0.01 and ****p*≤0.001 (Student’ s two-tailed *t*-test).

## Supplementary Tables

**Table S1**. Sequences of the native promoters used in this study.

| **Gene number** | **Gene**  **product** | **Sequence (5’-3’)** | **Lengthh**  **(bp)** |
| --- | --- | --- | --- |
| SACE_0009 | DNA gyrase subunit A | TGCTGATGCAGGTCACCCTCGACGACGCGGCGACGGCCGACGAGCTGTTCAGCGTGCTGATGGGCGAGGACGTCGAGGCCCGGCGCTCGTTCATCACCCGCAACGCCAAGGACGCGGGTCTGCTCGACATCTGACATCGCGGCCACCCGCCATCCCCGGAGGACCCCCGCCATGGGCAGTCGCCAGAAGTGCCCCGTTCGCAACGGAACGGCAAAGGTCCGAGGGAACACTCGCCCGCGTTGTGGTCGCGCAGGAACACGCTGACCCGCCTGAAAGGAACCCCCACCC | 288 |
| SACE_0029 | sigma-70 family RNA polymerase sigma factor | ATGGCCACCACCATCGGTTTCCACCGCGAGGTCGTCGACCACCCGGTTTTCCGCGACGGTGCCGCCGGCACGGACTTCGTGCGGCAGCTCCGCGACGGGTGACCCGGTCGGCGCGGCCCGGCTCTTTCTCGATCCCGAACGCGAACCAGTCATCGTGACCGATTTCCCGCGAATCTTCCGGCACCACCATCGAAGGTGATCAAGGTTCGAACGCCAGCCACCTGCCCGCGAGGAGAGTTGCCGGAGGGAGAACACG | 256 |
| SACE_0112 | sigma-70 family RNA polymerase sigma factor | AGGTTGCGGACCTTCGCCGACTTCTCGCTCGTGCTCACCGTGACGTGCCCGGCGTCGTCGGTGGTGGCCAGCACCGGCGACATCTGCGGTGTCCCGTCCGCGCGCAGGGTGCTGAGCACGGCCCGGTGCTGCTGCCGGACCACTGCCACCGCTTCTTCGAGATCCATGTTCGCCTCCCGTGAGCTCCGTCACCGGCATCCACTCGTCCGAGTGAGGCCGGAGTACCGATCTTCCCAGGTCACGCGATACCAAGTGGGTCGCCGAAACTGGGGAGGGCCATTC | 282 |
| SACE_0129 | SigE family RNA polymerase sigma factor | CTGGAGTCGCTGGACTCCGAGCCGCTCAACGCCGCCGAGCGGCGGTCCCGGGACGGTCTGCGCGGCAGGCAGATCACCGTCCGCTGAGTCACGGTTCGGCGGCGATCACCCCCACATTGCTCCGATCGAGTGACGAAGGGGGGCCGCGGCAACCGATCATGGTGGACGGACGTGTCCTCCACGGCGAGTCCCGGCGTGGAGGGAGTGGAGTGTGACCGCGCACCTGCCTGCTGCACCCGCATCCGCCGCGGAGGCGGCACGCAGCTGGCCTGGGCGCGCGCATCAGGGGGAC | 292 |
| SACE_0141 | sigma-70 family RNA polymerase sigma factor | TGTACCAACCAACCCGCAACAGCGGAAGGTCCCGGCGGGAAATCCGTCGCGGCGCCGGCCTGGAAACCGCCGCGGCACGGGATGCGTATTCCCTGGCAGATCCCGGCAAGAACCGCCGGGACGGCTCGAAGGGAACACCCATGCGCACCTCCACTCCGGCATCCTCCCGGCCTTCGCGCGCACCGGCACGGTCACCGTCCTCGACAGCGGGTAGGCGATCCGGAAAACCGTTCCGCGGTCCCGTGCGTCAGGAGTGTCGTGCGCGATCGAGAACTGAGCGACGAGCGGTTGCTCCGGAAA | 300 |
| SACE_0234 | RNA polymerase sigma factor | CTCACTCTGGGACGTGGTTCGCGACCGGCGGCTCAACCACCGCCCGGAGGTCGTGGGCGGGGTGCTCGAGGCGGAGTGCTCGGCGCTGCTGGAGGCCTTCTTCCACGGCCAGCGCTGACCCGCTCGGTGATCTTCGGGCGCCCCGGACAAGGGCCTTGCGGGGGCCGCGCATTCCACCGGTTCGGGTGGCCTGTCCCGGCCGTCATCCGATGCCCGGCCTGTGACGATGGATCCGCCCCGCCGGCGCGAGCGGGGCGGATCCATCGCACTTCGGGGGTTGGCGTGGAACCGACGGATGCT | 300 |
| SACE_0443 | Cysteinyl-tRNA synthetase | AGGTGCTGAGCAGGCTGCACTCCGACGGGGTGCGGATCGCGGTGATCAGCAACATCGCCTTCGACATCAGGCCGGCCTTCGAGAGGCTGGGCGTGACCGGACTCGTCGACGAGTTCGTGCTCTCCTACCTCGAAGGGGTGATCAAGCCCGATCCGAAGATCTTCCTGCGCGCCGCCGAGCGGGTCGGTGTCGAGCCCGGTGACGCGCTGATGATCGGTGACCACGCGGACGTCGACGGCGCGGCCGCGGCGGTCGGATGCCGGGTCGCCATCGTGGATCCGGCACCGACCTCCGAACGCCCGGACGCGCTGCTCACGGCGGTCGCCGAGCACGGTTTCTGACCGCTGGTCCCAGGCCGCCGACGTCCCTTCGGCGGAGTCCGGCGCGCACCGGGTGGAGGCACCCGGACGGGTGCCCGGGCGGGCGGTGCGGCGGAGGGAACCCGTTTTGGGCGTGCCCGTACCATCAGGGGC | 473 |
| SACE_1037 | RNA polymerase sigma factor SigE | ACATTGTTAGGCCCCCGTTTCCCGTCCCGACGTCCCTTAGCCCGATGGTGTGAGCTGCACATCACGACCCGTTCGGCGGTTCTCAGGCTGCTCTCAGGCACCTTTCATGGCCTCCTCACCAGCGCGGAGGAGATTGGTGACGCACAACCGGGCGTCTCCCGCTCCCGGCGGGTGACGCACCGCAGGAACAAAGCGACCGGTACGCACGTTCACCCTTGTGCAGCCGCCCGCATGGAGGTGGGTACCCGCCCGATGGCAACAAAGCCG | 267 |
| SACE_2041 | Aspartyl-tRNA synthetase | TGCTGGTCCCTGCCACACACCGCCCGCACCCGCTGTCGCGCCCACGTCTGCCTACCGCGCTGCGCCCGCTGTCGCGCATCGCCCGTTCGCCGCGCCGCTCACCGCCGAGCCCGCCTGCCGCGCCGCGCCGACTCGCCGCGCCTGCCTGCCGCGCCCGCCTCCTCACCGCCGAGCCCGCCTGCCGCGTCGCGCCGCCTCGCCGCGCCGCGCTCCCGGCCACCCCGTGGAGAGAAACCTGCTCGACGGCACCCGTGCCCCGTTGGCTAGCCTTGGAGCCTCGCGTCCTCGCTGCAGCGGCCCCACGAGCAAGGGTGCCGGCGCCGGGGCGCGCCACACACCGAACCACGCGCCGTCCACGTCAAGAGGAGCTCAGTACC | 378 |
| SACE_2101 | DNA-directed RNA polymerase subunit omega | AGTCCGAGTTCGACGCGACCGTGGTCAACGCCGACGTGCGGTCGGCGACCAGCGAATTGATAGGATTGGTGGTCGGCCGCCAACGGTGAGACCGTTCGACGCGGACCGGGGCGACCCGGTTCGCGCCGACGCGCAGCCGGACGGGGGCGGTCGGACGGCTGCCGCCGAGCGGACCACGCCCAGCGAGCCCGGCCCGCCAGGACCGGCCCCGGCCGGGCCTGGAGCCGGTCGGCACTGCCGGACGCCCGCCGCGCCCGTCGGCGCAAAGCCCGGCACCGGCACCGGTGCCCGTTCATTCACGAGGAGCAGGAGCCACTCAC | 321 |
| SACE_2226 | Cysteinyl-tRNA synthetase | TCACCCCGGCATGAGGAGACGCGGTGGATGTGCGCTGTTCGATCGTACGACCAGGTGGCGCTCCGGCATACTCGGAACTTCCGCGGACCGTATTCCGGTGAAATCGAACGGAAATGAGATCTGCAAAAGAATGGCGATTGGAGGGATGATCGGCGAAAGCGTTCTCCTCGCACGGGCCGCCGGGGCGGCGAGCACGACGACCACCTGTTCGGAGCACACGTGCGTCCCACTCTGTGATACGCACGATGGTTCCGGCGGGGGCATCCGGCCGGGGCCGACAGATAAGGTCGGGTG | 294 |
| SACE_5686 | RNA polymerase sigma factor SigF | ACACGCCCACGTCGAACCTGGTCGAGGTCCGCGTCGCTGCGGAGCCCGCCCAGCTCGCGGTGATGCGTGCGGTCATCGGCGACCTCGCCATGCGCGCGGATTTCGACGTCGACTCGATCGCCGACCTGCGGCTGGCAGTGGACGAGGCCTGCTCGTCGCTGGTCCGCCTCGCCGCTCCGTCGGCCTCCCTGGTCTGCCGGTTCCACATCCACGAGGGCGGGCTCTCGGTCACCGCGGAGGTGATCAGCGACGATGCCTTCGGCCCTCGCAAGGACACCTTCAGCTGGCGCGTGCTCAGCGCACTCGCCGACAGTGTGTCAACCTCGGTTCAGTCCGATGCGGGCTCCGACGGGAGCCATCTGGTGCGCATCGAACTGACCAAGGGACGGACGGCTCACGA | 400 |
| SACE_5720 | pyruvate kinase | ACAACACCGCGTAGGCCAGCAGGCCCGGCAGCAGCGGGCGACGCAGGCCCGCCATCCGGCCGGTGATCGCCAGCGCCGCGAACAGCAGCAGGTGCACGACCTTGTCGGTGCCGGGCGGCGCGACCGGAACGCCGGATTCGGGGGTGAACAGGACGACCAGGCTGGCCGCCACCGCCAGACCGAACGGAACCCTGGTGAGCTGCGCTCTGTAATGATCCAGCAACGATGTTCCCCGATTCGACTCCGCAGCCGACGCTGCGTACTGCACGCGGCGGTCAACAGACTAGGCTGACCCAAC | 298 |
| SACE_5882 | 30S ribosomal protein S12 methylthiot | TCTCGCCCGGCAGTGGGAACGTGCCGTGCGCACGGGGTTTCCGGTGCGACGCGGGGGTTTCGCCGCGGTTGCCGCGAGGCGTCCCGCGGGGCGGTTCCGTCCGCCGGATCCCGCCGGAGCCCGCCCGCGGTGCGCAACGAGGCTCATGCGCAGCGCGGACAACGCCGGACGTTACGCTCGACGC | 184 |
| SACE_6041 | RNA polymerase sigma factor WhiG | TCCGGTCCAGCGTTGTTCCTGGGCGCGCGAGGCGGCCGTCTTGATCCACGCGCGGTGCGTCGTGTCGTGCACGATGCGGTGGGGGCCGTTTCCGGTGCTACCGACGTGGGGCCGCACGGACTGCGTCACTCCGCGGCGACCCACTTACTCGAAGGAGGAGCGGACCTCCGTAGCGTTCAGGAGTTGCTTGGTCACGCTACGCTCGCAACGACGCAGCTTTACACACACGTGACCGTCGAACGGCTGAAGGCGATCC | 256 |
| SACE_6148 | Glutamyl and glutaminyl-tRNA synthetase | AGGTCGTCCATGAGCTGGAGGGTGGTCGGGTGCACCGTGTCGCCGCGGAAGTCGCGCAGGAAGTCGTTGTGCTTCTCCAGCACGGTGACCTCGACCCCCGCCCGGGCCAGCAGCAGTCCGAGGACCATGCCGGCGGGACCGCCGCCGACGATGCAACACGTCGTTCGCTCCATCGCGATCCTCCAAGTTTTCAACGCATGTTGAATAACTTCAGGGTGCCCTGCCGGGACTCGCCGGTCAAGGGCGGAAGCCGACTGGATACGCTGAACGCGTT | 274 |
| SACE_6592 | Tryptophanyl-tRNA synthetase | CGGTGACCCGCTCCCGGTCGGCGGGGTGAGCTTGCGAGAATCAGGACGTGAGTGACGCGCCCGCAACCCAGAACGCCGAGACGTCCGCGGCCCGGCCGCGC | 101 |
| SACE_662 | RNA polymerase sigma factor SigC | ACGAACAGCAGCACCGAGACCAGCATCCGGCGCCACAGGTCCCGGCTGCGGTCGCCGGAGTCGGGTTCCTCCGCGCTTTCCGGCGTCACCGGTTCCGCCTGGTAGCCGGCCCGTCGCACGGCGTCGAGCAGCGCCGCGTCGTCGGTGGCGGAAGCGACCTCGACGCTCGCGCGGCCCGTGGCGTAGTTCACGCTCGCCCGCACGCCGTCGAGCTTGCCGAGCTTGCGCTCCACGCGGGTCGCGCACGCCGCGCAGGTCATGCCGGTCACGGCGAGCTCCACCTGCCGCGCCTGCGGGCGCGGGGTAACGCGGGTCGGGGTCGGGGTCATCCGGGGCCTTTCAGCGTCGTGCGTGCCCGGCGGCGCTGTGGACGTCTCGACCGGGCTCTGCACAGGTGGTCGCATCGTGAGAACCACCGGTTCCCGGGCTCTTCGAGAGATCACGATCGAGGTA | 453 |
| SACE_6803 | RNA polymerase subunit alpha | TGTCGAAGTGGGACATCATCGACGTCAAGCAGAAGTCGCTGGGCACCACTCCGTTCATCATCGCCAAGGAGACCATCGGCGAGCGCCCGGTCCCGGCCTGGCTGCAGGTCGTGCCCTCGAACCTGCGGATCCTGGTGCACCAGCGCCCGGAGCGCGCGCAGATCGACACCCCGGTCACCGAGCAGCTCATCGTCGAGCTCTACTCGAAGTGACGCCTTCCGCCGGCGGCCACCACGTGGTCGCCGGCGGTGGCAGTCGCGGTGGTGGTGGGCCGATACCACCGCCACCGGCCCGCTTCGAGCGGGCACGCGTCCCATCGGCGTCAAATAGCGGGCGCCGTGAGGAAAGGAAACTCAA | 357 |
| SACE_6841 | 30S ribosomal protein S12 | AGACCGATCGGGTCGATCCGCATGCCCGTGCGTGACAGGTGACTGCGTGCAGCTCCATCGCGGTTCGCAGTCCCGGACACCGACGCGGGCGGCGGAACTCCTCCTGACAGCCCACTGGGGTCATTCCCGTTGGGACGCCATCAGCGCCGCAAGGCCGAGGAGCGGGCGACACGCCCGACCTCGTGGTGCGGGGGAGTCCCAAGTACACAGTGCCGCAGGTGCTCGACGCCAGGGGCACTGTGCGTCAAGCGGAGCTCGGACGAGCGGCGCTTGGCCGAGTACCCAGAAGACGCGAACGCTGACAGAAAGCCGGTCC | 316 |
| SACE_6853 | RNA polymerase subunit beta | GGAGATCACCGGGTTGCTGGCCTCGACCAAGCCCAACCGCGACGGCGAGCGGATGGTCAAGGCCAACGGCAAGGCCCAGCTGTTCGACGGGCGCAGCGGCGAGCCGTACCCGTACCCGGTGGCTGTCGGGTACATGTACATCCTCAAGCTGTCGCACCTGGTGGACGACAAGATCCACGCCCGGTCGACCGGTCCGTACTCGATGATCACCCAGCAGCCGCTGGGTGGTAAGGCGCAGTTCGGTGGCCAGCGCTTCGGTGAGATGGAGTGCTGGGCCATGCAGGCCTACGGCGCCGCGTACACGCTGCAGGAGCTGCTGACGATCAAGTCCGACGACGTGGTCGGCCGCGTCAAGGTCTACGAGGCGATCGTCAAGGGCGAGAACATCCCCGAGCCGGGCATCCCGGAGTCGTTCAAGGTGTTGCTCAAGGAGCTGCAGTCGCTGTGCCTGAACGTCGAGGTGCTCTCCAGCGACGGTGCCGCGATCGAGATGCGCGACGGTGACGACGAGGACCTGGAGCGTGCCGCGGCCAACCTCGGCATCAACCTGTCCCGCAACGAGTCGCCCTCCGTGGACGACGTCGTCCAATGACCTCGCCGCCGGTCCGCGTAGCCGAACTGCGCGGACCGGCGTCCGACCCAACCTGGCACCTGCTGCCGCCCGCCGGCAGCACCTTCCGACAAAGGGAGAAGACCCGAC | 700 |
| SACE_6854 | RNA polymerase subunit beta | GCGCCACGAGGGTGATCGAAGCGTGATCTCTTGACTGCGGGCCCGCTCGGGTTCACTCTGGTCGTCGACACGGGGCTGGATCGCCGTCGGCGGGTCCCCCTTGAGAGCTGGCGTCATTCCGGCTAGACTGCTACTTTGCGCTGCCCTCTTTCCGTCTACCCCGCATGGGGGTCGTAGGATCGTGGGCGCCATTGCACCCTTGACAGCCGTGTTAGCGGGCCGGGGTCGGCAGTCGAGCCGCCGGACCTCTTCGCTTTCTGATGCGACTGTAGCCAGTTCAGAGTCCCGGAAGGACGCATC | 300 |
| SACE_7382 | RNA polymerase sigma factor SigM | CGCGAGGTCGTCATCAACTCGCCGAGCGGCGGCGCGCGGGTCGAGATCCACAAGGTCGAGGGCGCGCCGTCGCCGAGCGCGGACACCCTGATCGGTTCCGGCAAGCTGGAGGCGGGCCGGACGCCGATCCAGATCGAGATGACCGGCAAGACCGAGAAGATCCTGATCGTCTTCACCCAGCTCGTGCCGGGTCCCGACGGCAGGTTCTCGTCGCAGATCAACGAGGTCACCGTCACCGGCTGATATCACTGGCGCAACGGGGCAGCCGGTGGGAGCCGGCGATCAGTAGAGTGCTCAATC | 300 |

**Table S2.** Promoter panel arranged according to the relative strengths in *S. erythraea* NRRL 23338. *p_ermE*_* strength was set to 1, and the fold changes represented the strengths of different promoters compared to *p_ermE*_*. Varied sequences of each promoter were indicated in red characters. -35 regions were indicated by solid lines, and -10 regions were indicated by dot lines.

| **Promoter name** | **Sequence (5’-3’)** | **Fold change** |
| --- | --- | --- |
| *p_ermE*-s43_* | TTGACGTGGTAGCGCATCGACACAGAGGAT | 0.31 |
| *p_ermE*-s42_* | TTGACGCTGCAGTATCTGGTGATGGAGGAT | 0.47 |
| *p_ermE*_* | TTGACGGCTGGCGAGAGGTGCGGGGAGGAT | 1.00 |
| *p_SACE_2101-S35_* | CGCATCCCGGCACCGGTGCCCGCACCAC | 1.07 |
| *p_ermE*-s41_* | TTGACGCCGGGATTTGGACTCAACGAGGAT | 1.22 |
| *p_SACE_2101-S43_* | AAGAAACCGGCACCGGTGCCCGGCGGCA | 1.98 |
| *p_SACE_2101-S33_* | GTCGCGCCGGCACCGGTGCCCGCACGCG | 3.15 |
| *p_ermE*-s23_* | TTGACGCTCTGGGGTTGTGAAGTAGAGGAT | 3.40 |
| *p_SACE_2101_* | CCGGCACCGGCACCGGTGCCCGTTCATT | 4.31 |
| *p_SACE_2101-S32_* | CGAGAGCCGGCACCGGTGCCCGTTTGGC | 6.68 |

**Table S3.** Top eight differently expressed *ery* genes in S0 compared to *S. erythraea* NRRL 23338.

| **Product** | **Gene** | **Relative transcriptional level (fold-change)** |
| --- | --- | --- |
| EryBIV | *SACE_0720* | 90.33334 |
| EryCI | *SACE_0734* | 72.09634 |
| EryBV | *SACE_0719* | 68.11421 |
| EryCIV | *SACE_0716* | 65.3893 |
| EryF | *SACE_0730* | 32.12651 |
| EryCVI | *SACE_0718* | 20.88467 |
| EryBVI | *SACE_0717* | 19.55096 |
| EryBIII | *SACE_0731* | 19.35683 |

**Table S4.** HPLC analysis of gene overexpressing strains.

| **Strain** | **Average peak area (A.U.)** | **Erythromycin (mg/L)** |
| --- | --- | --- |
| *S. erythraea* NRRL 23338 | 97.55929 | 2.098915 |
| *S. erythraea/*pSET152-hyg-egfp(ATG) | 93.00531 | 1.519242 |
| *S. erythraea/*pSET152-hyg-*p_SACE_2101_s32_*-*SACE_0716* | 188.378 | 15.84466 |
| *S. erythraea/*pSET152-hyg-*p_SACE_2101_s32_*-*SACE_0717* | 256.0066 | 25.83044 |
| *S. erythraea/*pSET152-hyg-*p_SACE_2101_s32_*-*SACE_0718* | 677.1088 | 88.00868 |
| *S. erythraea/*pSET152-hyg-*p_SACE_2101_s32_*-*SACE_0719* | 371.6934 | 36.99335 |
| *S. erythraea/*pSET152-hyg-*p_SACE_2101_s32_*-*SACE_0720* | 2054.146 | 251.152 |
| *S. erythraea/*pSET152-hyg-*p_SACE_2101_s32_*-*SACE_0731* | 541.8429 | 58.65161 |
| *S. erythraea/*pSET152-hyg- *p_ermE*_s23_*-*SACE_0716* | 170.0009 | 18.23774 |
| *S. erythraea/*pSET152-hyg- *p_ermE*_s23_*-*SACE_0717* | 175.0789 | 11.96636 |
| *S. erythraea/*pSET152-hyg- *p_ermE*_s23_*-*SACE_0718* | 163.6273 | 10.50869 |
| *S. erythraea/*pSET152-hyg- *p_ermE*_s23_*-*SACE_0719* | 338.5075 | 32.76912 |
| *S. erythraea/*pSET152-hyg- *p_ermE*_s23_*-*SACE_0720* | 1765.473 | 214.4071 |
| *S. erythraea/*pSET152-hyg- *p_ermE*_s23_*-*SACE_0731* | 2248.052 | 275.8344 |
| *S. erythraea/*pSET152-hyg-*p_SACE_2101_s32_*-*SACE_0718-p_ermE*_s23_*-*SACE_0719* | 2344.072 | 288.0567 |

**Table S5.** Strains and plasmids used in this study.

| **Strain and plasmid** | **Characteristics** | **Source** |
| --- | --- | --- |
| **Strain** |  |  |
| *Saccharopolyspora erythraea* NRRL 23338 | Wild-type *S. erythraea* strain of low erythromycin production | Lab stock |
| S0 | Industrial *S. erythraea* strain of high erythromycin production | Lab stock |
| *Escherichia coli* DH5α | Recipient strain used for plasmid construction | Lab stock |
| *Escherichia coli* ET12567/pUZ8002 | Helper strain used for conjugational transfer harboring plasmid pUZ8002, chloramphenicol and kanamycin resistant | Lab stock |
| *Escherichia coli* DB3.1 | Recipient strain used for constructing *ccdB*-containing plasmids | Lab stock |
| *S. erythraea/*pSET152-hyg-*p_SACE_0009_*-egfp(ATG) | *S. erythraea* haboring plasmid pSET152-hyg-*p_SACE_0009_*-egfp(ATG), hygromycin resistant | This study |
| *S. erythraea/*pSET152-hyg-*p_SACE_0029_*-egfp(ATG) | *S. erythraea* haboring plasmid pSET152-hyg hyg-*p_SACE_0029_*-egfp(ATG), hygromycin resistant | This study |
| *S. erythraea/*pSET152-hyg-*p_SACE_0112_*-egfp(ATG) | *S. erythraea* haboring plasmid pSET152-hyg hyg-*p_SACE_0112_*-egfp(ATG), hygromycin resistant | This study |
| *S. erythraea/*pSET152-hyg-*p_SACE_0129_*-egfp(ATG) | *S. erythraea* haboring plasmid pSET152-hyg-*p_SACE_0129_*-egfp(ATG), hygromycin resistant | This study |
| *S. erythraea/*pSET152-hyg-*p_SACE_0141_*-egfp(ATG) | *S. erythraea* haboring plasmid pSET152-hyg-*p_SACE_0141_*-egfp(ATG), hygromycin resistant | This study |
| *S. erythraea/*pSET152-hyg-*p_SACE_0234_*-egfp(ATG) | *S. erythraea* haboring plasmid pSET152-hyg-*p_SACE_0234_*-egfp(ATG), hygromycin resistant | This study |
| *S. erythraea/*pSET152-hyg-*p_SACE_0443_*-egfp(ATG) | *S. erythraea* haboring plasmid pSET152-hyg-*p_SACE_0443_*-egfp(ATG), hygromycin resistant | This study |
| *S. erythraea/*pSET152-hyg-*p_SACE_1037_*-egfp(ATG) | *S. erythraea* haboring plasmid pSET152-hyg-*p_SACE_1037_*-egfp(ATG), hygromycin resistant | This study |
| *S. erythraea/*pSET152-hyg-*p_SACE_2041_*-egfp(ATG) | *S. erythraea* haboring plasmid pSET152-hyg-*p_SACE_2041_*-egfp(ATG), hygromycin resistant | This study |
| *S. erythraea/*pSET152-hyg-*p_SACE_2101_*-egfp(ATG) | *S. erythraea* haboring plasmid pSET152-hyg-*p_SACE_2101_*-egfp(ATG), hygromycin resistant | This study |
| *S. erythraea/*pSET152-hyg-*p_SACE_2226_*-egfp(ATG) | *S. erythraea* haboring plasmid pSET152-hyg-*p_SACE_2226_*-egfp(ATG), hygromycin resistant | This study |
| *S. erythraea/*pSET152-hyg-*p_SACE_5686_*-egfp(ATG) | *S. erythraea* haboring plasmid pSET152-hyg-*p_SACE_5686_*-egfp(ATG), hygromycin resistant | This study |
| *S. erythraea/*pSET152-hyg-*p_SACE_5720_*-egfp(ATG) | *S. erythraea* haboring plasmid pSET152-hyg-*p_SACE_5720_*-egfp(ATG), hygromycin resistant | This study |
| *S. erythraea/*pSET152-hyg-*p_SACE_5882_*-egfp(ATG) | *S. erythraea* haboring plasmid pSET152-hyg-*p_SACE_5882_*-egfp(ATG), hygromycin resistant | This study |
| *S. erythraea/*pSET152-hyg-*p_SACE_6041_*-egfp(ATG) | *S. erythraea* haboring plasmid pSET152-hyg-*p_SACE_6041_*-egfp(ATG), hygromycin resistant | This study |
| *S. erythraea/*pSET152-hyg-*p_SACE_6148_*-egfp(ATG) | *S. erythraea* haboring plasmid pSET152-hyg -*p_SACE_6148_*-egfp(ATG), hygromycin resistant | This study |
| *S. erythraea/*pSET152-hyg-*p_SACE_6592_*-egfp(ATG) | *S. erythraea* haboring plasmid pSET152-hyg-*p_SACE_6592_*-egfp(ATG), hygromycin resistant | This study |
| *S. erythraea/*pSET152-hyg-*p_SACE_6625_*-egfp(ATG) | *S. erythraea* haboring plasmid pSET152-hyg-*p_SACE_6625_*-egfp(ATG), hygromycin resistant | This study |
| *S. erythraea/*pSET152-hyg-*p_SACE_6803_*-egfp(ATG) | *S. erythraea* haboring plasmid pSET152-hyg-*p_SACE_6803_*-egfp(ATG), hygromycin resistant | This study |
| *S. erythraea/*pSET152-hyg-*p_SACE_6841_*-egfp(ATG) | *S. erythraea* haboring plasmid pSET152-hyg-*p_SACE_6841_*-egfp(ATG), hygromycin resistant | This study |
| *S. erythraea/*pSET152-hyg-*p_SACE_6853_*-egfp(ATG) | *S. erythraea* haboring plasmid pSET152-hyg-*p_SACE_6853_*-egfp(ATG), hygromycin resistant | This study |
| *S. erythraea/*pSET152-hyg-*p_SACE_6854_*-egfp(ATG) | *S. erythraea* haboring plasmid pSET152-hyg-*p_SACE_6854_*-egfp(ATG), hygromycin resistant | This study |
| *S. erythraea/*pSET152-hyg-*p_SACE_7382_*-egfp(ATG) | *S. erythraea* haboring plasmid pSET152-hyg-*p_SACE_7382_*-egfp(ATG), hygromycin resistant | This study |
| *S. erythraea/*pSET152-hyg-*p_SACE_2101_s32_*-*SACE_0716* | *S. erythraea* haboring plasmid pSET152-hyg-*p_SACE_2101_s32_*-*SACE_0716*, hygromycin resistant | This study |
| *S. erythraea/*pSET152-hyg-*p_SACE_2101_s32_*-*SACE_0717* | *S. erythraea* haboring plasmid pSET152-hyg-*p_SACE_2101_s32_*-*SACE_0717*, hygromycin resistant | This study |
| *S. erythraea/*pSET152-hyg-*p_SACE_2101_s32_*-*SACE_0718* | *S. erythraea* haboring plasmid pSET152-hyg-*p_SACE_2101_s32_*-*SACE_0718*, hygromycin resistant | This study |
| *S. erythraea/*pSET152-hyg-*p_SACE_2101_s32_*-*SACE_0719* | *S. erythraea* haboring plasmid pSET152-hyg-*p_SACE_2101_s32_*-*SACE_0719*, hygromycin resistant | This study |
| *S. erythraea/*pSET152-hyg-*p_SACE_2101_s32_*-*SACE_0720* | *S. erythraea* haboring plasmid pSET152-hyg-*p_SACE_2101_s32_*-*SACE_0720*, hygromycin resistant | This study |
| *S. erythraea/*pSET152-hyg-*p_SACE_2101_s32_*-*SACE_0731* | *S. erythraea* haboring plasmid pSET152-hyg-*p_SACE_2101_s32_*-*SACE_0731*, hygromycin resistant | This study |
| *S. erythraea/*pSET152-hyg-*p_ermE*_s23_*-*SACE_0716* | *S. erythraea* haboring plasmid pSET152-hyg-*p_ermE*_s23_*-*SACE_0716*, hygromycin resistant | This study |
| *S. erythraea/*pSET152-hyg-*p_ermE*_s23_*-*SACE_0717* | *S. erythraea* haboring plasmid pSET152-hyg-*p_ermE*_s23_*-*SACE_0717*, hygromycin resistant | This study |
| *S. erythraea/*pSET152-hyg-*p_ermE*_s23_*-*SACE_0718* | *S. erythraea* haboring plasmid pSET152-hyg-*p_ermE*_s23_*-*SACE_0718*, hygromycin resistant | This study |
| *S. erythraea/*pSET152-hyg-*p_ermE*_s23_*-*SACE_0719* | *S. erythraea* haboring plasmid pSET152-hyg-*p_ermE*_s23_*-*SACE_0719*, hygromycin resistant | This study |
| *S. erythraea/*pSET152-hyg-*p_ermE*_s23_*-*SACE_0720* | *S. erythraea* haboring plasmid pSET152-hyg-*p_ermE*_s23_*-*SACE_0720*, hygromycin resistant | This study |
| *S. erythraea/*pSET152-hyg-*p_ermE*_s23_*-*SACE_0731* | *S. erythraea* haboring plasmid pSET152-hyg-*p_ermE*_s23_*-*SACE_0731*, hygromycin resistant | This study |
| *S. erythraea/*pSET152-hyg-*p_SACE_2101_s32_*-*SACE_0718-p_ermE*_s23_*-*SACE_0719* | *S. erythraea* haboring plasmid pSET152-hyg-*p_SACE_2101_s32_*-*SACE_0718-p_ermE*_s23_*-*SACE_0719*, hygromycin resistant | This study |
| **Plasmid** |  |  |
| pSET152-hyg | Integrated plasmid, hygromycin resistant | Lab stock |
| pSET152-hyg-*p_SACE_0009_*-egfp(ATG) | Integrated plasmid with *p_SACE_0009_*-egfp reporter gene, hygromycin resistant | This study |
| pSET152-hyg-*p_SACE_0029_*-egfp(ATG) | Integrated plasmid with *p_SACE_0029_*-egfp reporter  gene, hygromycin resistant | This study |
| pSET152-hyg-*p_SACE_0112_*-egfp(ATG) | Integrated plasmid with *p_SACE_0112_*-egfp reporter gene, hygromycin resistant | This study |
| pSET152-hyg-*p_SACE_0129_*-egfp(ATG) | Integrated plasmid with *p_SACE_0129_*-egfp reporter gene, hygromycin resistant | This study |
| pSET152-hyg-*p_SACE_0141_*-egfp(ATG) | Integrated plasmid with *p_SACE_0141_*-egfp reporter gene, hygromycin resistant | This study |
| pSET152-hyg-*p_SACE_0234_*-egfp(ATG) | Integrated plasmid with *p_SACE_0234_*-egfp reporter gene, hygromycin resistant | This study |
| pSET152-hyg-*p_SACE_0443_*-egfp(ATG) | Integrated plasmid with *p_SACE_0443_*-egfp reporter gene, hygromycin resistant | This study |
| pSET152-hyg-*p_SACE_1037_*-egfp(ATG) | Integrated plasmid with *p_SACE_1037_*-egfp reporter gene, hygromycin resistant | This study |
| pSET152-hyg-*p_SACE_2041_*-egfp(ATG) | Integrated plasmid with *p_SACE_2041_*-egfp reporter gene, hygromycin resistant | This study |
| pSET152-hyg-*p_SACE_2101_*-egfp(ATG) | Integrated plasmid with *p_SACE_2101_*-egfp reporter gene, hygromycin resistant | This study |
| pSET152-hyg-*p_SACE_2226_*-egfp(ATG) | Integrated plasmid with *p_SACE_2226_*-egfp reporter gene, hygromycin resistant | This study |
| pSET152-hyg-*p_SACE_5686_*-egfp(ATG) | Integrated plasmid with *p_SACE_5686_*-egfp reporter gene, hygromycin resistant | This study |
| pSET152-hyg-*p_SACE_5720_*-egfp(ATG) | Integrated plasmid with *p_SACE_5720_*-egfp reporter gene, hygromycin resistant | This study |
| pSET152-hyg-*p_SACE_5882_*-egfp(ATG) | Integrated plasmid with *p_SACE_5882_*-egfp reporter gene, hygromycin resistant | This study |
| pSET152-hyg-*p_SACE_6041_*-egfp(ATG) | Integrated plasmid with *p_SACE_6041_*-egfp reporter gene, hygromycin resistant | This study |
| pSET152-hyg-*p_SACE_6148_*-egfp(ATG) | Integrated plasmid with *p_SACE_6148_*-egfp reporter gene, hygromycin resistant | This study |
| pSET152-hyg-*p_SACE_6592_*-egfp(ATG) | Integrated plasmid with *p_SACE_6592_*-egfp reporter gene, hygromycin resistant | This study |
| pSET152-hyg-*p_SACE_6625_*-egfp(ATG) | Integrated plasmid with *p_SACE_6625_*-egfp reporter gene, hygromycin resistant | This study |
| pSET152-hyg-*p_SACE_6803_*-egfp(ATG) | Integrated plasmid with *p_SACE_6803_*-egfp reporter gene, hygromycin resistant | This study |
| pSET152-hyg-*p_SACE_6841_*-egfp(ATG) | Integrated plasmid with *p_SACE_6841_*-egfp reporter gene, hygromycin resistant | This study |
| pSET152-hyg-*p_SACE_6853_*-egfp(ATG) | Integrated plasmid with *p_SACE_6853_*-egfp reporter gene, hygromycin resistant | This study |
| pSET152-hyg-*p_SACE_6854_*-egfp(ATG) | Integrated plasmid with *p_SACE_6854_*-egfp reporter gene, hygromycin resistant | This study |
| pSET152-hyg-*p_SACE_7382_*-egfp(ATG) | Integrated plasmid with *p_SACE_7382_*-egfp reporter gene, hygromycin resistant | This study |
| pSET152-hyg-*p_SACE_2101_*-egfp(ATG)-ccdB | Integrated helper plasmid in golden-gate assembly harboring *ccdB* gene, hygromycin resistant | This study |
| pSET152-hyg*-p_ermE*_*-egfp(ATG)-ccdB | Integrated helper plasmid in golden-gate assembly harboring *ccdB* gene, hygromycin resistant | This study |
| pSET152-hyg-*p_SACE_2101_*- (1035 lib)-egfp(ATG) | Integrated plasmid with *p_SACE_2101_* promoter library (-10 and -35 regions mutated), hygromycin resistant | This study |
| pSET152-hyg-*p_ermE*_*-spacer(lib)-egfp(ATG) | Integrated plasmid with *p_ermE*_* promoter library (spacer region mutated), hygromycin resistant | This study |
| pSET152-hyg-*p_2101_s32_*-*SACE_0716* | Integrative plasmid harboring *p_2101_s32_* and *SACE_0716*, hygromycin resistant | This study |
| pSET152-hyg-*p_2101_s32_*-*SACE_0717* | Integrative plasmid harboring *p_2101_s32_* and *SACE_0717*, hygromycin resistant | This study |
| pSET152-hyg-*p_2101_s32_*-*SACE_0718* | Integrative plasmid harboring *p_2101_s32_* and *SACE_0718*, hygromycin resistant | This study |
| pSET152-hyg-*p_2101_s32_*-*SACE_0719* | Integrative plasmid harboring *p_2101_s32_* and *SACE_0719*, hygromycin resistant | This study |
| pSET152-hyg-*p_2101_s32_*-*SACE_0720* | Integrative plasmid harboring *p_2101_s32_* and *SACE_0720*, hygromycin resistant | This study |
| pSET152-hyg-*p_2101_s32_*-*SACE_0731* | Integrative plasmid harboring *p_2101_s32_* and *SACE_0731*, hygromycin resistant | This study |
| pSET152-hyg-*p_ermE*_s23_*-*SACE_0716* | Integrative plasmid harboring *p_ermE*_s23_* and *SACE_0716*, hygromycin resistant | This study |
| pSET152-hyg-*p_ermE*_s23_*-*SACE_0717* | Integrative plasmid harboring *p_ermE*_s23_* and *SACE_0717*, hygromycin resistant | This study |
| pSET152-hyg-*p_ermE*_s23_*-*SACE_0718* | Integrative plasmid harboring *p_ermE*_s23_* and *SACE_0718*, hygromycin resistant | This study |
| pSET152-hyg-*p_ermE*_s23_*-*SACE_0719* | Integrative plasmid harboring *p_ermE*_s23_* and *SACE_0719*, hygromycin resistant | This study |
| pSET152-hyg-*p_ermE*_s23_*-*SACE_0720* | Integrative plasmid harboring *p_ermE*_s23_* and *SACE_0720*, hygromycin resistant | This study |
| pSET152-hyg-*p_ermE*_s23_*-*SACE_0731* | Integrative plasmid harboring *p_ermE*_s23_* and *SACE_0731*, hygromycin resistant | This study |
| pSET152-hyg-*p_2101_s32_*-*SACE_0718-p_ermE*_s23_*-*SACE_0719* | Integrative plasmid harboring *p_2101_s32_*、*SACE_0718、p_ermE*_s23_* and *SACE_0719*, hygromycin resistant | This study |

**Table S6.** Primes used in this study.

| **Purpose** | **Primer name** | **Sequence (5’-3’)** | | |
| --- | --- | --- | --- | --- |
| **Promoter mining and engineering** |  |  | | |
| pSET152-hyg-*p_SACE_0009_*-egfp(ATG) | SACE_0009-F | CCAGTGCCAAGCTTGGGCTGTGCTGATGCAGGTCACCCTCG | | |
|  | SACE_0009-R | TCCTCGCCCTTGCTGACCATGGGTGGGGGTTCCTTTCAGG | | |
| pSET152-hyg-*p_SACE_0029_*-egfp(ATG) | SACE_0029-F | CCAGTGCCAAGCTTGGGCTGATGGCCACCACCATCGGTTT | | |
|  | SACE_0029-R | TCCTCGCCCTTGCTGACCATCGTGTTCTCCCTCCGGCAACT | | |
| pSET152-hyg-*p_SACE_0112_*-egfp(ATG) | SACE_0112-F | CCAGTGCCAAGCTTGGGCTGAGGTTGCGGACCTTCGCCGA | | |
|  | SACE_0112-R | TCCTCGCCCTTGCTGACCATGAATGGCCCTCCCCAGTTTC | | |
| pSET152-hyg-*p_SACE_0129_*-egfp(ATG) | SACE_0129-F | CCAGTGCCAAGCTTGGGCTGCTGGAGTCGCTGGACTCCGA | | |
|  | SACE_0129-R | TCCTCGCCCTTGCTGACCATGTCCCCCTGATGCGCGCGCC | | |
| pSET152-hyg-*p_SACE_0141_*-egfp(ATG) | SACE_0141-F | CCAGTGCCAAGCTTGGGCTGTGTACCAACCAACCCGCAAC | | |
|  | SACE_0141-R | TCCTCGCCCTTGCTGACCATTTTCCGGAGCAACCGCTCGT | | |
| pSET152-hyg-*p_SACE_0234_*-egfp(ATG) | SACE_0234-F | CCAGTGCCAAGCTTGGGCTGCTCACTCTGGGACGTGGTTC | | |
|  | SACE_0234-R | TCCTCGCCCTTGCTGACCATAGCATCCGTCGGTTCCACGC | | |
| pSET152-hyg-*p_SACE_0443_*-egfp(ATG) | SACE_0443-F | CCAGTGCCAAGCTTGGGCTGAGGTGCTGAGCAGGCTGCAC | | |
|  | SACE_0443-R | TCCTCGCCCTTGCTGACCATGCCCCTGATGGTACGGGCAC | | |
| pSET152-hyg-*p_SACE_1037_*-egfp(ATG) | SACE_1037-F | CCAGTGCCAAGCTTGGGCTGACATTGTTAGGCCCCCGTTT | | |
|  | SACE_1037-R | TCCTCGCCCTTGCTGACCATCGGCTTTGTTGCCATCGGGC | | |
| pSET152-hyg-*p_SACE_2041_*-egfp(ATG) | SACE_2041-F | CCAGTGCCAAGCTTGGGCTGTGCTGGTCCCTGCCACACAC | | |
|  | SACE_2041-R | TCCTCGCCCTTGCTGACCATGGGTACTGAGCTCCTCTTGA | | |
| pSET152-hyg-*p_SACE_2101_*-egfp(ATG) | SACE_2101-F | CCAGTGCCAAGCTTGGGCTGAGTCCGAGTTCGACGCGACC | | |
|  | SACE_2101-R | TCCTCGCCCTTGCTGACCATGTGAGTGGCTCCTGCTCCTC | | |
| pSET152-hyg-*p_SACE_2226_*-egfp(ATG) | SACE_2226-F | CCAGTGCCAAGCTTGGGCTGTCACCCCGGCATGAGGAGAC | | |
|  | SACE_2226-R | TCCTCGCCCTTGCTGACCATCACCCGACCTTATCTGTCGG | | |
| pSET152-hyg-*p_SACE_5686_*-egfp(ATG) | SACE_5686-F | CCAGTGCCAAGCTTGGGCTGACACGCCCACGTCGAACCTG | | |
|  | SACE_5686-R | TCCTCGCCCTTGCTGACCATTCGTGAGCCGTCCGTCCCTT | | |
| pSET152-hyg-*p_SACE_5720_*-egfp(ATG) | SACE_5720-F | CCAGTGCCAAGCTTGGGCTGACAACACCGCGTAGGCCAGCA | | |
|  | SACE_5720-R | TCCTCGCCCTTGCTGACCATGTTGGGTCAGCCTAGTCTGT | | |
| pSET152-hyg-*p_SACE_5882_*-egfp(ATG) | SACE_5882-F | CCAGTGCCAAGCTTGGGCTGTCTCGCCCGGCAGTGGGAAC | | |
|  | SACE_5882-R | TCCTCGCCCTTGCTGACCATGCGTCGAGCGTAACGTCCGG | | |
| pSET152-hyg-*p_SACE_6041_*-egfp(ATG) | SACE_6041-F | CCAGTGCCAAGCTTGGGCTGTCCGGTCCAGCGTTGTTCCT | | |
|  | SACE_6041-R | TCCTCGCCCTTGCTGACCATGGATCGCCTTCAGCCGTTCG | | |
| pSET152-hyg-*p_SACE_6148_*-egfp(ATG) | SACE_6148-F | CCAGTGCCAAGCTTGGGCTGAGGTCGTCCATGAGCTGGAG | | |
|  | SACE_6148-R | TCCTCGCCCTTGCTGACCATAACGCGTTCAGCGTATCCAG | | |
| pSET152-hyg-*p_SACE_6592_*-egfp(ATG) | SACE_6592-F | CCAGTGCCAAGCTTGGGCTGCGGTGACCCGCTCCCGGTCG | | |
|  | SACE_6592-R | TCCTCGCCCTTGCTGACCATGCGCGGCCGGGCCGCGGACGTCTC | | |
| pSET152-hyg-*p_SACE_6625_*-egfp(ATG) | SACE_6625-F | CCAGTGCCAAGCTTGGGCTGACGAACAGCAGCACCGAGAC | | |
|  | SACE_6625-R | TCCTCGCCCTTGCTGACCATTACCTCGATCGTGATCTCTC | | |
| pSET152-hyg-*p_SACE_6803_*-egfp(ATG) | SACE_6803-F | CCAGTGCCAAGCTTGGGCTGTGTCGAAGTGGGACATCATC | | |
|  | SACE_6803-R | TCCTCGCCCTTGCTGACCATTTGAGTTTCCTTTCCTCACG | | |
| pSET152-hyg-*p_SACE_6841_*-egfp(ATG) | SACE_6841-F | CCAGTGCCAAGCTTGGGCTGAGACCGATCGGGTCGATCCG | | |
|  | SACE_6841-R | TCCTCGCCCTTGCTGACCATGGACCGGCTTTCTGTCAGCG | | |
| pSET152-hyg-*p_SACE_6853_*-egfp(ATG) | SACE_6853-F | CCAGTGCCAAGCTTGGGCTGGGAGATCACCGGGTTGCTGG | | |
|  | SACE_6853-R | TCCTCGCCCTTGCTGACCATGTCGGGTCTTCTCCCTTTGT | | |
| pSET152-hyg-*p_SACE_6854_*-egfp(ATG) | SACE_6854-F | CCAGTGCCAAGCTTGGGCTGGCGCCACGAGGGTGATCGAA | | |
|  | SACE_6854-R | TCCTCGCCCTTGCTGACCATGATGCGTCCTTCCGGGACTC | | |
| pSET152-hyg-*p_SACE_7382_*-egfp(ATG) | SACE_7382-F | CCAGTGCCAAGCTTGGGCTGCGCGAGGTCGTCATCAACTC | | |
|  | SACE_7382-R | TCCTCGCCCTTGCTGACCATGATTGAGCACTCTACTGATC | | |
| The vector backbone of the constructed plasmid above | 152egfp(ATG)-F | ATGGTCAGCAAGGGCGAGGA | | |
|  | pSET152-R | CAGCCCAAGCTTGGCACTGG | | |
| pSET152-hyg-*p_SACE_2101_* -egfp(ATG)-ccdB | p2101(lib)-ccdB-F | GCCGCGCCCGTCGGCGCACATGAGACCACGCGTGGATCCGGCTTACT | | |
|  | p2101(lib)-ccdB-R | TCCTCGCCCTTGCTGACCATGTGATGAGACCTTATATTCCCCAGAACATCA | | |
|  | p2101(lib)-bb-R | TGTGCGCCGACGGGCGCGGC | | |
|  | lib-bb-F | ATGGTCAGCAAGGGCGAGG | | |
| pSET152-hyg-*p_ermE*_* -egfp(ATG)-ccdB | permE(lib)-ccdB-F | CGCAGGTGCACGCGGTCGATTGAGACCACGCGTGGATCCGGCTTACT | | |
|  | permE(lib)-ccdB-R | TCCTCGCCCTTGCTGACCATGTGGTGAGACCTTATATTCCCCAGAACATCA | | |
|  | ermE(lib)-bb-R | ATCGACCGCGTGCACCTGCG | | |
|  | lib-bb-F | ATGGTCAGCAAGGGCGAGG | | |
| pSET152-hyg-*p_SACE_2101_* -1035( lib)-egfp(ATG) | p2101(lib)_1035 -F | CCAGGTCTCACACAAGCNNNNNNCCGGCACCGGTGCCCGNNNNNNCACGAGGAGCAGGAGCCACTC | | |
|  | p2101(lib)_1035 -R | TCCTCGCCCTTGCTGACCATGTGAGTGGCTCCTGCTCCTC | | |
| pSET152-hyg-*p_ermE*_* -spacer(lib)-egfp(ATG) | permE(lib)_spacer -F | CCAGGTCTCACGATCTTGACGNNNNNNNNNNNNNNNNNNGAGGATCTGACCGACGCGG | | |
|  | permE(lib)_spacer -R | CCAGGTCTCAGTGGTGTCCTACCAACCGGC | | |
| **Gene overexpression** |  | | |  |
| The vector backbone of the overexpressing plasmid | 152-bb-F | | | CGCGGCCGCGCGCGATATCGAATTC |
|  | 152GBD-bb-R | | | GCACTGGCCGTCGTTTTACAACGTCGTGACTGGGAAAACC |
| pSET152-hyg-*p_2101_s32_*-*SACE_0716* | 152GBD-A-0716-F | | | GAGGAGCAGGAGCCACTCACATGAAACGCGCGCTGACCGA |
|  | 152GBD-A-0716-R | | | GAATTCGATATCGCGCGCGGCCGCGTCACGAACCGTTGCGCGTCC |
| pSET152-hyg-*p_2101_s32_*-*SACE_0717* | 152GBD-A-0717-F | | | GAGGAGCAGGAGCCACTCACGTGCGGGTCTTGATCGACAA |
|  | 152GBD-A-0717-R | | | GAATTCGATATCGCGCGCGGCCGCGTCATCCGGCGGTCCTGGTGTA |
| pSET152-hyg-*p_2101_s32_*-*SACE_0718* | 152GBD-A-0718-F | | | GAGGAGCAGGAGCCACTCACATGTACGAGGGCGGGTTCGCCGAGCTTTACGACC |
|  | 152GBD-A-0718-R | | | GAATTCGATATCGCGCGCGGCCGCGTCATCCGCGCACACCGACGA |
| pSET152-hyg-*p_2101_s32_*-*SACE_0719* | 152GBD-A-0719-F | | | GAGGAGCAGGAGCCACTCACGTGCGGGTACTGCTGACGTC |
|  | 152GBD-A-0719-R | | | CGAATTCGATATCGCGCGCGGCCGCGCTAGCCGGCGTGGCGGCGCGTG |
| pSET152-hyg-*p_2101_s32_*-*SACE_0720* | 152GBD-A-0720-F | | | GAGGAGCAGGAGCCACTCACGTGAATGGGATCAGTGATTC |
|  | 152GBD-A-0720-R | | | GAATTCGATATCGCGCGCGGCCGCGCTAGTGCTCCTCGGTGGGGGTCAGGGCGGCCA |
| pSET152-hyg-*p_2101_s32_*-*SACE_0731* | 152GBD-A-0731-F | | | GAGGAGCAGGAGCCACTCACATGATCTTCCTTGTGGGACT |
|  | 152GBD-A-0731-R | | | GAATTCGATATCGCGCGCGGCCGCGTCATACGACTTCCAGTCGGGGTAGGGGGA |
| pSET152-hyg-*p_ermE*_s23_*-*SACE_0716* | 152GBD-eA-0716-F | | | GCCGGTTGGTAGGACACCACATGAAACGCGCGCTGACCGA |
|  | 152GBD-eA-0716-R | | | GAATTCGATATCGCGCGCGGCCGCGTCACGAACCGTTGCGCGTCC |
| pSET152-hyg-*p_ermE*_s23_*-*SACE_0717* | 152GBD-eA-0717-F | | | GCCGGTTGGTAGGACACCACGTGCGGGTCTTGATCGACAACG |
|  | 152GBD-eA-0717-R | | | GAATTCGATATCGCGCGCGGCCGCGTCATCCGGCGGTCCTGGTGT |
| pSET152-hyg-*p_ermE*_s23_*-*SACE_0718* | 152GBD-eA-0718-F | | | GCCGGTTGGTAGGACACCACATGTACGAGGGCGGGTTCGCCGAGCTTTAC |
|  | 152GBD-eA-0718-R | | | GAATTCGATATCGCGCGCGGCCGCGTCATCCGCGCACACCGACGA |
| pSET152-hyg-*p_ermE*_s23_*-*SACE_0719* | 152GBD-eA-0719-F | | | GCCGGTTGGTAGGACACCACGTGCGGGTACTGCTGACGTC |
|  | 152GBD-eA-0719-R | | | GAATTCGATATCGCGCGCGGCCGCGCTAGCCGGCGTGGCGGCGCGTGAGTTCCTCCAGTC |
| pSET152-hyg-*p_ermE*_s23_*-*SACE_0720* | 152GBD-eA-0720-F | | | GCCGGTTGGTAGGACACCACGTGAATGGGATCAGTGATTCCCCG |
|  | 152GBD-eA-0720-R | | | GAATTCGATATCGCGCGCGGCCGCGCTAGTGCTCCTCGGTGGGGGTCAGGGCGGCCACC |
| pSET152-hyg-*p_ermE*_s23_*-*SACE_0731* | 152GBD-eA-0731-F | | | GCCGGTTGGTAGGACACCACATGATCTTCCTTGTGGGACTAGGC |
|  | 152GBD-eA-0731-R | | | GAATTCGATATCGCGCGCGGCCGCGTCATACGACTTCCAGTCGGGGT |
| cloning the mutant promoter-  *p_2101_s32_* | M13-F | | | TGTAAAACGACGGCCAGTGC |
|  | SACE_2101-R1 | | | GTGAGTGGCTCCTGCTCCTC |
| cloning the mutant promoter-  *p_ermE*_s23_* | M13-F | | | TGTAAAACGACGGCCAGTGC |
|  | ermE*-R1 | | | GTGGTGTCCTACCAACCGGC |
| pSET152-hyg-*p_2101_s32_*-*SACE_0718-p_ermE*_s23_*-*SACE_0719* | ZH-152-3218-bb-R | | | TGCTCGGGTCGGGCTGGTACCTCATCCGCGCACACCGACGA |
|  | ZH-23-0719-ermE-F | | | GGTACCAGCCCGACCCGAGCA |
| **qRT-PCR** |  | |  | |
| Gene-SACE_0713 | SACE_0713-F | | TGGCCGAACGGATCGCGGAT | |
|  | SACE_0713-R | | GCCAGGACAAGGCGGGAGAT | |
| Gene-SACE_0714 | SACE_0714-F | | TAGTCGGGCAGCATCCCTCG | |
|  | SACE_0714-R | | AGCACGCCGTACGTGGCCGAGT | |
| Gene-SACE_0715 | SACE_0715-F | | TTCGGGTTGTCCGTGGGCAC | |
|  | SACE_0715-R | | ATGTTCCGCTGCCACTTCTGC | |
| Gene-SACE_0716 | SACE_0716-F | | GCGAACGTCATCGAAGGCATGA | |
|  | SACE_0716-R | | ACTGCGTGGCCACCTGCAAC | |
| Gene-SACE_0717 | SACE_0717-F | | AGCGCGGCACGTTCAACACG | |
|  | SACE_0717-R | | AGCTGGCGCCTACGGTGCACT | |
| Gene-SACE_0718 | SACE_0718-F | | GCCACGACCCAGTGGATCTCCA | |
|  | SACE_0718-R | | TTCCTCGACGGCTACGTGGC | |
| Gene-SACE_0719 | SACE_0719-F | | GTTCTCGGTGTAGGTGGGGTC | |
|  | SACE_0719-R | | GATTGCATGCTACGCGGCCAG | |
| Gene-SACE_0720 | SACE_0720-F | | TCGGTCTTCTGCTGCGCGTACC | |
|  | SACE_0720-R | | AACGTCGGCCTGATGCACGACC | |
| Gene-SACE_0721 | SACE_0721-F | | GTGTTGTCGGAGGTGGCCGGAT | |
|  | SACE_0721-R | | AACGGCACCGCAGGCACGCCACAA | |
| Gene-SACE_0722 | SACE_0722-F | | TTGGTCTTGGCTTTGCGGCGGT | |
|  | SACE_0722-R | | AGGCGAGAAGATCGCCAACC | |
| Gene-SACE_0723 | SACE_0723-F | | GCTGCTGCTGGAGACGAGCT | |
|  | SACE_0723-R | | TGTCCTCGCCGTAGCCGAAC | |
| Gene-SACE_0724 | SACE_0724-F | | GGGTTCTCCCTCGATCTCGT | |
|  | SACE_0724-R | | AGCCGGGTGAACTCGTGCGGA | |
| Gene-SACE_0725 | SACE_0725-F | | GAGATGACCGCGAACACCGTGG | |
|  | SACE_0725-R | | TCCAGGTGCAATGCCGGGTG | |
| Gene-SACE_0726 | SACE_0726-F | | AGTGGCTCACCTGGACGCTGGAG | |
|  | SACE_0726-R | | AGGCCGGTGTCGAGCCTGAT | |
| Gene-SACE_0727 | SACE_0727-F | | GGCAAGGTCTCCTACGTCGGCT | |
|  | SACE_0727-R | | ACCGCCAGGTTGTACAGGCACT | |
| Gene-SACE_0728 | SACE_0728-F | | TGGAGGAGGCCAACCAGGAG | |
|  | SACE_0728-R | | CTTGCGCGTCTCGAGCCAGA | |
| Gene-SACE_0729 | SACE_0729-F | | AGTGCTGCAACCAGCGCTCG | |
|  | SACE_0729-R | | ACTACCGCGTGCTGCGCTCCTA | |
| Gene-SACE_0730 | SACE_0730-F | | ACCGCCGATCTCCACCTCCT | |
|  | SACE_0730-R | | ACCTACCTGCTGCTCACCCA | |
| Gene-SACE_0731 | SACE_0731-F | | ACGTTGCCCTTGGTCGAGGC | |
|  | SACE_0731-R | | ACACCATGGCCCCCTACGAG | |
| Gene-SACE_0732 | SACE_0732-F | | AACACCGTCGTGGTGCTCAACACCG | |
|  | SACE_0732-R | | TCGCCGAACAGCAGCGCCGT | |
| Gene-SACE_0733 | SACE_0733-F | | GGGTGCTGCCGATCCTGGTT | |
|  | SACE_0733-R | | CGTGCCGGTTGGTAGGATCCA | |
| Gene-SACE_0734 | SACE_0734-F | | TTCAGCTTCTACCCGGGCAAG | |
|  | SACE_0734-R | | ACCTCGTGCACGTACTTCTG | |
| Reference gene | sigA (1801)-F | | TCTTGGCCGCAGAACTCTTG | |
|  | sigA (1801)-R | | TCCAGCTCCGCTGCAAACTC | |
